# Temperate Mountain Forest Biodiversity under Climate Change: Compensating Negative Effects by Increasing Structural Complexity

**DOI:** 10.1371/journal.pone.0097718

**Published:** 2014-05-13

**Authors:** Veronika Braunisch, Joy Coppes, Raphaël Arlettaz, Rudi Suchant, Florian Zellweger, Kurt Bollmann

**Affiliations:** 1 Forest Research Institute of Baden-Württemberg, Freiburg, Germany; 2 Conservation Biology, Institute of Ecology and Evolution, University of Bern, Bern, Switzerland; 3 Swiss Ornithological Institute, Valais Field Station, Sion, Switzerland; 4 Swiss Federal Institute for Forest, Snow and Landscape Research, Birmensdorf, Switzerland; 5 Institute of Terrestrial Ecosystems, Swiss Federal Institute of Technology, Zürich, Switzerland; Key Laboratory of Tropical Forest Ecology, Xishuangbanna Tropical Botanical Garden, Chinese Academy of Sciences, China

## Abstract

Species adapted to cold-climatic mountain environments are expected to face a high risk of range contractions, if not local extinctions under climate change. Yet, the populations of many endothermic species may not be primarily affected by physiological constraints, but indirectly by climate-induced changes of habitat characteristics. In mountain forests, where vertebrate species largely depend on vegetation composition and structure, deteriorating habitat suitability may thus be mitigated or even compensated by habitat management aiming at compositional and structural enhancement. We tested this possibility using four cold-adapted bird species with complementary habitat requirements as model organisms. Based on species data and environmental information collected in 300 1-km^2^ grid cells distributed across four mountain ranges in central Europe, we investigated (1) how species’ occurrence is explained by climate, landscape, and vegetation, (2) to what extent climate change and climate-induced vegetation changes will affect habitat suitability, and (3) whether these changes could be compensated by adaptive habitat management. Species presence was modelled as a function of climate, landscape and vegetation variables under current climate; moreover, vegetation-climate relationships were assessed. The models were extrapolated to the climatic conditions of 2050, assuming the moderate IPCC-scenario A1B, and changes in species’ occurrence probability were quantified. Finally, we assessed the maximum increase in occurrence probability that could be achieved by modifying one or multiple vegetation variables under altered climate conditions. Climate variables contributed significantly to explaining species occurrence, and expected climatic changes, as well as climate-induced vegetation trends, decreased the occurrence probability of all four species, particularly at the low-altitudinal margins of their distribution. These effects could be partly compensated by modifying single vegetation factors, but full compensation would only be achieved if several factors were changed in concert. The results illustrate the possibilities and limitations of adaptive species conservation management under climate change.

## Introduction

With a predicted global temperature increase of 2.0–4.5°C until the end of the century (IPPC 2007), climate change is expected to affect habitat quality and species distributions [1]. Impacts have been demonstrated for all continents and taxonomic groups [2], [3], however, geographically isolated species adapted to cold climatic conditions [4] face a particularly high risk of range contractions, if not local extinction [5], [6]. In Europe, adverse effects are therefore mainly predicted for boreo-alpine taxa of mountain ecosystems [7], often being glacial relicts occurring at the margins of their eco-climatic niche [8], [9]. Species range-shifts are usually predicted based on large-scale species distribution models [10], describing species presence as a function of current climatic variation as well as coarse-grained, area-wide available environmental data [11], [12]. Yet, the populations of many endothermic species may not be primarily affected by physiological constraints of climate warming, but indirectly by climate-induced changes in habitat quality, food availability or interspecific interactions [1]. Consequently, the validity of predictions merely relying on climate functions may be questioned.

In forest ecosystems, biodiversity largely depends on the diversity of forest composition and structure (e.g., variability in tree species composition, vertical and horizontal forest structure, age structure of the stands, presence of gaps, clearings, snags and dead wood) [13], [14]. Next to the site conditions [15], the structural characteristics of montane and subalpine forests are mainly attributed to cold ambient temperatures which entail low forest productivity, long succession cycles and a high potential for snow-break or wind-throw with subsequent susceptibility for insect calamities [16], [17]. Although natural stand dynamics and resulting structural attributes are largely overruled by forestry, climate change is expected to affect forest vegetation composition and structure, and consequently, habitat suitability and distribution of the associated species. Adverse effects may therefore be additionally amplified by forestry practices aimed at coping with the economic risks of climate change such as the shortening of harvesting periods or changes in the tree species portfolio. On the other hand, a species’ dependence on vegetation characteristics may also offer the opportunity to counter negative effects of climate change by targeted habitat management (e.g., by increasing particular, species-relevant structural elements or vegetation components). We tested this option using the example of four mountain bird species of conservation concern: capercaillie (*Tetrao urogallus*), hazel grouse (*Bonasa bonasia*), three-toed woodpecker (*Picoides tridactylus*) and pygmy owl (*Glaucidium passerinum*). These species have been proposed as indicators for different, complementary forest structural attributes, and represent different niche dimensions within the mountain forest ecosystem. Capercaillie and three-toed woodpecker are additionally regarded as umbrella species for the associated ecological communities [18]–[20], thus supporting our aim to evaluate management measures that may support a wider range of biodiversity in mountain forests.

The model species show a high degree of specialization which facilitates tracking their responses to vegetation structures and climate-related variation thereof. The capercaillie is considered as an indicator for structurally rich, boreal and mountain forest habitats [21]–[23]. These habitats are characterised by an intermediate canopy cover, high proportions of old and open stands, and abundant ground vegetation – ideally dominated by bilberry (*Vaccinium myrtillus*) [21], [24], [25]. Similar to capercaillie, the hazel grouse requires structurally rich stands [26], [27], but prefers younger successional stages with sufficient berry or catkin bearing trees and shrubs [26]–[30]. A dense understory of shrubs and herbs further provides summer foraging habitat and cover from predators for both ground-nesting grouse species [26], [27], [29], [31], [32]. By excavating cavities, the three-toed woodpecker provides breeding opportunities for a variety of cavity-breeding birds and bats [33] and is therefore considered a key-stone species [18], [34]. It mainly feeds on the larvae of bark and wood-boring insects, predominantly found in dying and dead conifer (mostly spruce) trees [35]–[37]. Dead trees, snags and dying trees are therefore one of the most important habitat features for foraging [33], [36], [38]–[42]. The pygmy owl is the smallest avian predator in European boreal and mountain forests [43], [44]. It hunts small mammals as well as birds, insects and reptiles [45], [46] and uses cavities created by woodpeckers as nesting places as well as to hoard food [46]–[48]. A combination of dense young stands with high cover and open old forest with some small clearings is considered good breeding habitat [49]. Inner forest edges and edges between successional stages are often used for hunting [49], [50].

All four species are listed in Annex 1 of the European Birds directive [51] and are thus frequently targeted by conservation and restoration programmes. With climate change, there is not only an emergent risk that the benefits of these programmes will be curtailed; the prevailing predictions of range contractions and local extinctions have also led to a general debate in conservation management and policy that fundamentally questions the effectiveness and possibility of preserving climatically vulnerable species in their current habitats [52], [53].

We address these questions by assessing (1) how the occurrence of our model species’ depends on climate, landscape and vegetation characteristics, (2) how climate change and associated vegetation changes will affect overall habitat suitability, and (3) if decisive habitat features could be modified by adaptive management in a way that negative effects of climate change could be mitigated or compensated. While being aware of the high susceptibility of climate-change-related forecasts to various sources such as variations in climate change scenarios [54], statistical methods [55], [56] and model parameterizations [57], [58], which we have evaluated for our model species in an earlier study [47], we do not aim to provide absolute measures of habitat suitability and their changes. Rather, focusing on one method and scenario of climate change as an example, we aim to provide rough estimates for the magnitude of both effects and management efforts that would be necessary to preserve the model species’ in their Central European mountain habitats, thereby evaluating the general possibilities and limitations of adaptive conservation management in mountain forest environments under climate change.

## Methods

### Ethics Statement

Species data were adopted from existing databases, thus no mapping or handling of endangered species was involved. Vegetation mapping was mostly conducted in state and public forests where no permits for were required. Access to communal and private forest in Germany was covered by the Federal Forest Law of Baden-Württemberg (LWaldG §74 section 1), which allows entering private property for research purposes; in Switzerland similar rights were given by the Swiss Forest Law (WaG, article 14, §1). Permits for vegetation mapping within protected areas in Baden-Württemberg, Germany were issued by the Regional Council Freiburg (Regierungspräsidium Freiburg) department for nature conservation, in Switzerland permits were given by the cantonal departments of forestry and the Swiss National Park administration. The coordinates of the study locations are provided in Table S1, with the grid cells entirely or partly located in protected areas indicated and the approving authorities specified.

### Study Area

**Figure 1:**
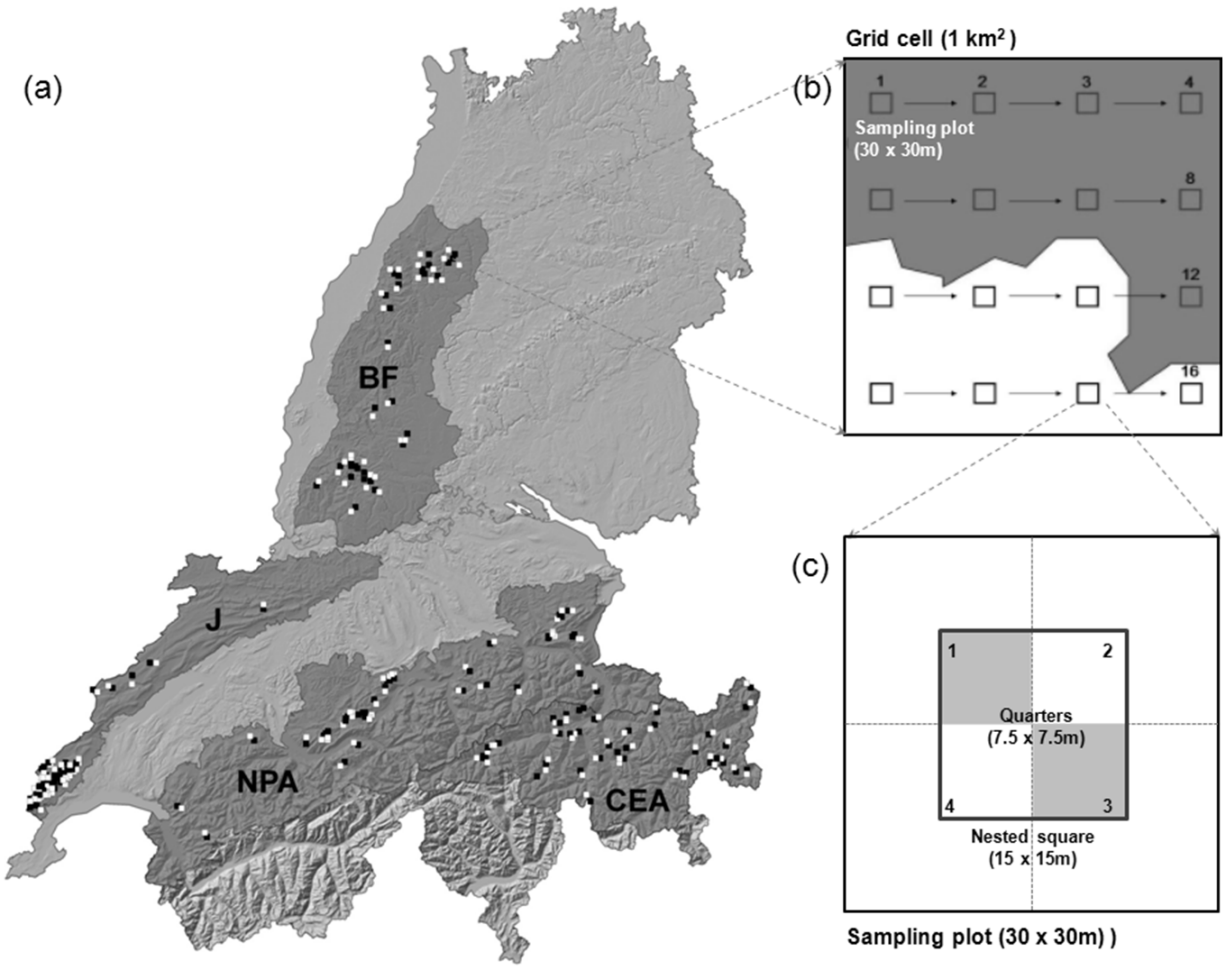
Study area (a) with the four mountain ranges [Black Forest (BF), Swiss Jura (J), Northern Prealps (NPA) and Central Eastern Alps (CEA)] and the spatial distribution of 1 km^2^ grid cells with species’ presence (white) and absence (black). Within each grid cell, environmental variables were recorded at or in the surrounding of maximum 16 regularly distributed sampling plots (b), with only plots located in the forest (dark grey) considered in the analysis. At each plot, vegetation variables were recorded in the field at different reference areas (c), either across the whole plot (30×30 m), within a nested square (15×15 m), or within the two diagonal quarters of which (7.5×7.5 m). The variables and the reference area at which they were recorded are specified in Table 2. Geodata: Switzerland: © Bundesamt für Landestopografie Swisstopo (Art. 30 GeoIV): License No.: 5704 000 000, Available at: http://www.swisstopo.admin.ch/internet/swisstopo/en/home/products/height/dhm25.html; Germany: © Landesamt für Geoinformation und Landentwicklung Baden-Württemberg (LGL), License No.: 2851.9-1/19, Avaliable at: http://www.lgl-bw.de/lgl-internet/opencms/de/07_Produkte_und_Dienstleistungen/Geodaten/Digitale_Gelaendemodelle.

The study area encompassed four mountain regions in Switzerland and Southern Germany with sympatric occurrence of the four model species, representing a broad gradient as regards climatic, vegetation and land-use conditions. The Black Forest, expanding over 7′000 km^2^ in Southwestern Germany, is a mainly forested lower mountain range with elevations ranging from 120 to 1′493 m a.s.l (mean: 663). The Swiss Jura, 4′200 km^2^ in size, is located in Western Switzerland and covers an altitudinal range between 500–1′718 m a.s.l (mean: 817). The Swiss Alps are here represented by two climatically and geographically distinct study regions: the “Northern Prealps”, defined by the biogeographic regions Prealps and Northern Alps with altitudes between 370–4′227 m a.s.l. (mean 1′391), and the Eastern Central Alps, with altitudes from 560–4′010 m a.s.l. (mean: 2′112) [59] (Figure 1). In the Black Forest and the Swiss Jura, where elevations do not reach the tree line, the forests form semi-continuous habitats interspersed by pasture land, while in the Northern Prealps forests surround treeless mountain tops. Finally, in the Eastern Central Alps, forests form distinct belts around high elevation peaks. Forest composition also varies along the altitudinal and climatic gradient, with decreasing proportions of European beech (*Fagus sylvatica*) and silver fir (*Abies alba*) giving way to a predominance of Norway spruce (*Picea abies*) when moving from the submontane to the subalpine belt. Moreover, larger proportions of larch (*Larix decidua*) and Swiss stone pine (*Pinus cembra*) can be found towards the Eastern Central Alps where a continental climate prevails in contrast to the other three regions, which are characterized by more oceanic climate conditions.

### Species Data

Data of species presence were adopted from two databases hosted by the Swiss Ornithological Institute, Sempach, Switzerland (http://www.ornitho.ch) and the Forest Research Institute of Baden-Württemberg (FVA), Germany (http://www.wildtiermonitoring.de). Both databases contain long-term collections of observation data from ornithologists, foresters, hunters, birdwatchers as well as research personnel at a minimum resolution of 1 km^2^. Since data were not sampled systematically, no proven absence data were available.

In each of the four study regions we selected at least ten 1 km^2^ grid cells for each of the four focal species with species observations in at least three years between 2006 and 2010 (Table 1, Figure 1). Presence cells were selected by a stratified random process so as to represent the extent of the species distribution and its climatic gradient in the respective study region, thereby preferring cells with repeated observations from multiple years. For each presence grid cell, a corresponding cell in the following referred to as “absence cell” was selected, with “absence” defined as cells with no recorded species proof within the preceding 11 years (2000–2010). Absence cells were selected within a maximum of 5 km distance to the presence cell by randomly choosing one of the surrounding cells with at least 50% forest cover, while excluding all cells directly adjacent to the presence cell. With this we ensured that absence cells were located within the species’ dispersal ranges and did not expand too far beyond the limits of their altitudinal-climatic range so as to avoid trivial results and unsubstantiated extrapolations. We used a similar number of grid cell-pairs for each species, yet, since the species were not equally distributed across the study region, the relative numbers and the spatial distributions of cells differed in the four study regions (Table 1, Figure S1 a–d).

**Table 1:** Number of grid cell pairs (1 km^2^) with species presence and absence selected in each of the mountain regions across the study area (BF: Black Forest, J: Swiss Jura, NPA: Northern Prealps, CEA: Central Eastern Alps).

Species	BF	J	NPA	CEA	Total
Capercaillie	23	21	16	11	71
Hazel grouse	0	28	27	13	68
Three-toed woodpecker	11	12	30	15	68
Pygmy owl	15	22	21	13	71

### Environmental Variables

#### Sampling scheme

Environmental predictors were sampled at 16 sampling plots, regularly distributed within each grid cell, with only plots located in the forest considered for the analysis (Figure 1). Our predictor set included variables of three main classes: climate, landscape and vegetation, measured at different reference areas around each sampling plot (Table 2).

**Table 2:** Variables used as predictors to model species presence, their source and the reference area at which they were recorded. Sources of the geodata (a–k) are provided in Appendix S1.

Category	Variable	Description	Unit	Reference area	Source
**Climate**					
	TEMPS	Average temperature in earlysummer (May–July)	°C	100×100 m	Wordclim/WSL^a^
	TEMPW	Average temperature in winter(Dec.–Feb.)	°C	100×100 m	Wordclim/WSL^a^
	PRECS	Precipitation sum May–July	mm	100×100 m	Wordclim/WSL^a^
	PRECW	Precipitation sum Dec.–Feb.	mm	100×100 m	Wordclim/WSL^a^
**Landscape**					
**Topography**	SLOPE	Slope	degree	30×30 m	DEM^b,c^
	TOPEX	Topographic position index	index	1 km^2^	DEM^b,c^
	EAST	Eastness (sine of aspect)	(−1)–1	30×30 m	DEM^b,c^
	NORTH	Northness (cosine of aspect)	(−1)–1	30×30 m	DEM^b,c^
	SOLAR57	Pot. solar radiation May–July	Wh/m^2^	30×30 m	DEM^b,c^
**Land cover**	FOREST	Forest	%	1 km^2^	Vektor25^d^/ATKIS^e^
	EDGEOUT	Density of outer forest edges	m/km^2^	1 km^2^	Vektor25^d^/ATKIS^e^
	FEDGEIN	Density of inner forest edges	m/km^2^	1 km^2^	Vektor25^d^/ATKIS^e^
	INTENSIVE	Intensive grassland and arableland	%	1 km^2^	GEOSTAT^f^/Landsat5^g^/
	EXTENSIVE	Extensive grassland	%	1 km^2^	GEOSTAT^f^/Landsat5^g^/
	WETSOIL	Proportion of mires and wetsoils	%	1 km^2^	Mire inventory BAFU^h^, FVA^i^ Vector25^d^/ATKIS^e^
**Infrastructure**	ROADDENS	Density of roads	m/km^2^	1 km^2^	Vektor25^d^/ATKIS^e^
	SETTLEDIST	Distance to settlements	m	Plot center	Vektor25^d^/ATKIS^e^
**Vegetation**					
**Vegetation structure**					
**Stand mosaic**	CHEIGHT2	Percentage of forest of height	%	1 km^2^	LiDAR^j,k^
	CHEIGHT3	classes 2,3 and 4, respectively			
	CHEIGHT4	2: <5 m			
		3: 5–15 m			
		4: >15 m			
	GAPINDEX	Number of gaps of at least0.1 ha	n/ha	1 km^2^	LiDAR^j,k^
	CHH	Canopy height heterogeneity:total edge length betweenheight classes 2, 3 and 4.	m/ha	1 km^2^	LiDAR^j,k^
	ED134	Length of “sharp” edges(between non-forested areas andforest of >5 m)	m/ha	1 km^2^	LiDAR^j,k^
	ED12	Length of “soft edges” (betweennon-forested areas and forest<5 m)	m/ha	1 km^2^	LiDAR^j,k^
**Stand structure**	SUCC	Age of the forest in 6 categories:1 = regeneration (<1.3 m height)2 = thicket (<10 cm DBH*)	Category 1–6	30×30 m	Fieldwork
		3 = pole stage (<30 cm DBH)			
		4 = tree stage (<60 cm DBH)			
		5 = „old“ forest (≥3tr. >60 cmDBH) 6 = multi-age			
					
	STANDSTRU	Vertical structure as number oflayers:	Category 1–3	30×30 m	Fieldwork
		1 = one,			
		2 = two			
		3 = multi layered			
	GVDIS	The pattern of ground vegetationwas classified into 3 categories:1 = homogeneous, 2 = patchy,3 = clumped	Category 1–3	30×30 m	Fieldwork
	CANCOV	Canopy (≥5 m) cover	%	30×30 m	Fieldwork
	SHRUBCOV	Shrub (≥1.3 m<5 m) cover	%	30×30 m	Fieldwork
	GVCOV	Ground vegetation (<1.3 m)cover	%	30×30 m	Fieldwork
**Vegetation composition**					
**Tree species**	BEE	Percent of beech	%	30×30 m	Fieldwork
	SPR	Percent of spruce	%	30×30 m	Fieldwork
	PIN	Percent of pine	%	30×30 m	Fieldwork
	FIR	Percent of fir	%	30×30 m	Fieldwork
	RESTREE	Percent of resource trees *(Sorbus*sp., *Salix* sp., *Betula* sp., *Alnus* sp.,*Corylus* sp. and *Sambucus* sp.)	%	30×30 m	Fieldwork
**Ground vegetation**	HERB	Percent of herbs	%	7.5×7.5 m	Fieldwork
	FERN	Percent of ferns	%	7.5×7.5 m	Fieldwork
	GRASS	Percent of fir grass	%	7.5×7.5 m	Fieldwork
	VAC	Percent of bilberry (Vaccinium sp)	%	7.5×7.5 m	Fieldwork
	BERRY	Percent of berries (other thanVaccinium sp.)	%	7.5×7.5 m	Fieldwork
**Special features**	ROW	Number of rowans >3 m	n	15×15 m	Fieldwork
	BBTREE	Number of basal branched trees	n	30×30 m	Fieldwork
	STANDDEAD	Number of standing dead trees >12 cm	n	30×30 m	Fieldwork
	HSTUMP	Number of hard stumps >12 cm	n	15×15 m	Fieldwork
	SSTUMP	Number of soft stumps >12 cm	n	15×15 m	Fieldwork
	E1	Presence of inner forest edgeecotone	1/0	30×30 m	Fieldwork
	E2	Presence of outer forest edgeecotone	1/0	30×30 m	Fieldwork

#### Climate

Climate variables included the average temperature in the breeding season (May–July) and in winter (December–February), and the sum of precipitation in both periods (Table 2). Current climate (long-term averages from 1971 to 2000) was obtained from the worldclim-dataset [60] (http://www.worldclim.org), which was downscaled from a 1 km^2^ raster to a resolution of 100×100 m based on the SRTM-V4 digital elevation model and the method described in [61].

For future climate conditions in the year 2050 (long-term averages from to 2031 to 2050) we assumed the moderate IPCC emission scenario A1B. Variables were derived from the Global Circulation Model ECHAM5, which was downscaled using the CLM Regional Circulation Model of the Max Planck Institute (http://cera-www.dkrz.de). A resolution of 100×100 m was then obtained by adding the anomalies between current and future climate conditions, which were downscaled to 1 km^2^ using the change factor methodology [62] to the current baseline data. All climate data were processed and provided by the Research Unit ‘Landscape Dynamics’ of the Swiss Federal Research Institute WSL.

#### Landscape

Landscape variables included information on topography, land cover and human infrastructure, obtained from different digital data sources (Table 2). Five topographical variables (slope, topographic position, eastness, northness and potential solar radiation) were derived from the digital elevation model (DEM) for each sampling plot. The topographic position index, calculated with the extension TPI 1.3a for ArcView 3.3 [63], qualifies a point’s position relative to the surrounding terrain, with negative values indicating exposed sites such as hilltops or ridges, and positive values representing depressions. The potential solar radiation [W*h/m^2^] in the breeding season was calculated according to Fu and Rich [64] using the function “area solar radiation” in ArcGIS 9.3 [65]. Land cover variables encompassed the proportion of forest cover, intensively and extensively used agricultural land and wetland (mires and other habitat types on wet soils), as well as the density of outer forest edges, within the surrounding area of 1 km^2^. Human infrastructure was represented by the density of trafficable roads per km^2^ and the distance to settlements.

#### Vegetation

Vegetation variables included information on vegetation composition (tree species and ground vegetation), vegetation structure (related to stand structure and forest stand mosaic) and special habitat features or resources relevant to the focal species. Vegetation composition, stand structure and special resources were mapped in the field at the sampling plots, while information on the forest stand mosaic was derived from remote sensing data. For matter of precision, different variables were assessed at different reference areas around the sampling plot center: tree species composition, successional stage, vertical and horizontal stand structure and selected special features (e.g., basal-branched trees or snags) were recorded within squares of 30×30 m, whereas special resources like the number of rowans or lying dead wood were quantified within a nested square of 15×15 m, the two diagonal quarters of which (7.5×7.5 m) were used to assess the ground vegetation (Figure 1, Table 2).

We derived variables describing the forest stand mosaic based on first and last return Light Detection and Ranging (LiDAR) data. For the cells in the Black Forest we used the revised point clouds for both terrain and surface models, as described in Schleyer [66], for Switzerland the corresponding data were provided by Swisstopo (2011). MATLAB R2011a (Mathworks, Natick, Massachusetts, USA) routines [67] were used to obtain terrain-corrected vegetation heights at a resolution of 3×3 m, which resembles the crown projection of a small spruce tree. The normalized vegetation heights were interpolated to form a continuous canopy height model, which was classified into four height classes: non-forested areas, shrub layer (<5 m), midstory (5–15 m) and canopy layer (>15 m) (processing details are provided in [68]). We used the height classes to calculate structural metrics describing the proportion of each height class per 1 km^2^, the number of gaps, the length of edges between different height classes representing different ecotone-types, as well as the total edge length between all height classes which provided an index for overall canopy height heterogeneity (for details see Table 2). Stand mosaic metrics were calculated in FRAGSTATS [48].

### Statistical Analysis

#### Species occurrence

We modelled species presence as a function of the environmental variables recorded at the sampling plots using Mixed Effects Logistic Regression with the grid-cell pair, as our species-sampling unit, treated as a random effect to account for spatial clustering. To identify the variables that best explained species presence we applied an information-theoretic approach [69], [70] using Akaike’s Information Criterion (AIC) to identify the most parsimonious model.

We followed a hierarchical variable selection procedure: first, univariate models were run for each variable, testing also the quadratic term for variables for which we expected a unimodal response. Of pairs of correlated variables (Spearman’s r ≥|0.6|) significantly contributing to explaining species presence in the univariate models we discarded the least performing one.

**Figure 2:**
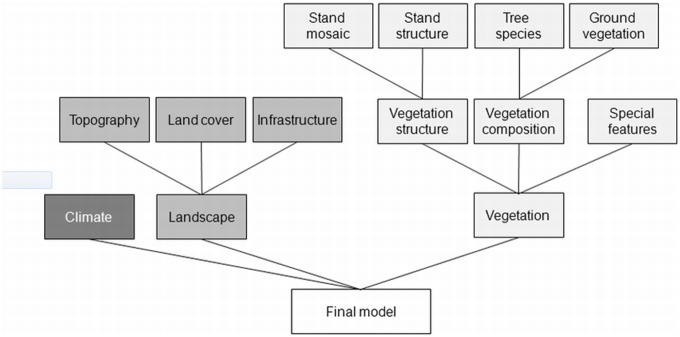
Hierarchical model selection process with arrows indicating the modelling steps: the variables were grouped into ecologically or functionally distinct variable subsets, for each of which a model was calibrated. The variables that significantly contributed to the most parsimonious model were retained for model calibration at the next hierarchy-level until a final model was obtained.

The retained predictors were then grouped into ecologically meaningful variable subsets (Figure 2, Table 2). For each subset a model was calibrated by testing all possible variable combinations and identifying the most parsimonious model using the R-package MuMIN. The variables that significantly contributed to this “best” subset-model were used for calibrating the model at the next hierarchy-level. This way the variable set was refined in a stepwise fashion, until a final model was obtained, potentially containing variables of all variable classes. The models’ fit was evaluated using multiple evaluation metrics, i.e., sensitivity, specificity, the percent correctly classified and Cohen’s Kappa at the optimal threshold, as well as the area under the receiver operating characteristics (ROC) curve (AUC).

#### Climate-related vegetation trends

To detect and quantify relationships between vegetation variables and climate, and obtain rough estimates of the magnitude and direction of possible vegetation changes under climate change, we used the data of all sampling plots mapped during the study (N = 4752) for modelling the species-relevant vegetation variables as functions of climate using Multiple Linear Regression. Only uncorrelated (Spearman’s r≥|0.6|) climate predictors that significantly contributed to explaining the respective vegetation characteristics were included. We applied these models to future climate conditions to calculate the mean expected change for each variable, which was then used to modify the respective variable value at each sampling plot to simulate vegetation changes under climate change.

#### Predicted changes in habitat suitability

The bird species’ models were extrapolated to future climate conditions and the predicted change in occurrence probability (as a surrogate for habitat suitability) was quantified. We first calculated the change in occurrence probability due to climate change alone holding the vegetation variables constant, and secondly, also included the modelled climate-induced changes of the vegetation.

#### Compensation potential

To evaluate the possibility to compensate for negative effects of climate change through habitat management, we selected for each species those among the significant vegetation variables that could potentially be modified by forest management. We then predicted species occurrence in dependence of these variables under current and future climate conditions while holding all other variables constant at their sampling average. Thereby each variable was only allowed to vary within the range of the empirical sampling values so as to avoid unfounded extrapolations outside the actually observed conditions. The comparison of the two response curves under current and future conditions illustrates the magnitude of variable modification that would be necessary for maintaining the current probability of species occurrence under the selected scenario of climate change. For each variable we then calculated the “compensation potential”, which was defined as the maximally achievable increase in predicted probability of species presence under altered climate conditions, which could be obtained when modifying the respective variable. Finally, to illustrate the effect of combining different measures of structural enhancement, we simultaneously varied the two variables for which the compensation potential was highest.

## Results

### Species’ Occurrence

The final models performed good to excellent [71] in predicting the presence of the model species. Accuracy was highest for the pygmy owl (AUC: 0.947, SD: 0.005) and lowest for the three-toed woodpecker (AUC: 0.877, SD: 0.010). The models for capercaillie and hazel grouse also demonstrated an excellent fit (AUC: 0.931 and 0.918, SD: 0.006 and 0.008, respectively). Further evaluation results are given in Table S2.

The final models of all four species contained variables of all main variable groups (climate, landscape, vegetation) (Table 3, Table S3). While all species showed a similar habitat selection pattern regarding climate, greater divergence was found for the decisive landscape variables and a different, even complementary set of vegetation and forest structural variables was retained for the different species (Table 3, Table S3). All species showed a unimodal response to winter temperature and a positive correlation with precipitation in early summer; in pygmy owl areas with higher winter precipitation were also selected. Concerning landscape characteristics, all species, except hazel grouse, preferred mires and forests on wet soils and showed at least a trend to avoid forests with a high road density and located in the vicinity of settlements. A negative response was also found for capercaillie towards a high density of outer forest edges, which can serve as an indicator for forest fragmentation. The presence of hazel grouse and three-toed woodpecker was negatively affected by the proportion of intensively managed agricultural land in the surroundings, while pygmy owl showed a positive correlation.

**Table 3:** Variables selected in the final models for capercaillie (CC), hazel grouse (HG) three-toed woodpecker (TTW) and pygmy owl (PO).

Category	Variable	CC	HG	TTW	PO
**Climate**	TEMPW	−−−	−−−	−−−	−−−
	TEMPW∧2	−	−	−	−−−
	PRECS	+++	++	+++	+++
	PRECW		n.s.		+++
**Landscape**	EAST	++			+
	SLOPE	n.s.			−
	SOLAR		+++		
	WETSOIL	++		+++	+++
	INTENSIVE		−−	−−−	++
	FEDGEOUT	−−−			
	FEDGEIN	n.s.			
	ROADDENS	−−−	n.s.		−−−
	SETTLEDIST	+	n.s.		+++
**Vegetation**	CHEIGHT4	+++	+	+++	+++
	CHEIGHT4∧2	−−−			
	GAPINDEX	+++			
	CHH	−−−			
	ED134				+++
	STANDSTRU 2			−−	
	STANDSTRU 3			−−	
	GVDIS (2: patchy)	n.s.	+		
	GVDIS (3: clumped)	n.s.	n.s.		
	SHRUBCOV			−	
	GVCOV				+
	BEE	−−−			
	BEE∧2	++			
	SPR			+++	
	SPR∧2			+++	
	PIN			+++	
	RESTREE		+	+	
	HERB	n.s.	++		
	FERN		n.s.		
	VAC	++	+++		
	STANDDEAD			++	
	HSTUMP	−−		−−	
	ROW	n.s.	n.s.		
	BBTREE		+		++
	E1	n.s.			
	E2		−		

The signs indicate a positive (+) or negative (−) correlation with species presence, their number specifies the significance level (+++/−−− p<0.001, ++/−− p<0.01, +/− p<0.05). For variable codes see Table 2, for detailed results see Table S3.

Except for the proportion of forest patches of the highest height class, which showed a quadratic relationship for capercaillie and was positively correlated with the presence of the three other species, the retained vegetation variables varied greatly between species’ models. Capercaillie presence was mainly explained by the abundance of gaps per km^2^, a low to moderate proportion of beech in the canopy and high cover of *Vaccinium* sp., mainly bilberry (*Vaccinium myrtillus*) in the field layer, as well as by low canopy height heterogeneity. Hazel grouse presence was mainly related to the availability of food sources, i.e. the proportion of resource trees and a high cover of herbs and bilberry; features providing cover, like basal-branched trees and a patchy ground vegetation distribution were preferred, while the vicinity of outer forest edges was avoided. Three-toed woodpecker occurrence was positively correlated with the presence of conifers and resource trees, and a high abundance of snags; while two- or multi-layered stands and stands with a high shrub cover were avoided. As in capercaillie, woodpecker presence was also negatively correlated with the abundance of hard stumps, indicating recent harvesting activities. Finally, pygmy owl habitat was characterized by a greater density of “sharp” forest edges: a greater abundance of basal-branched trees and higher ground vegetation cover than in locations where the species was absent.

### Climate-related Vegetation Trends

Although most study sites were located in managed forests, all species-relevant vegetation variables were significantly correlated with climate, which explained between 2% (percentage of forest with canopy height >15 m) and 21% (percentage of pine, *Pinus sp.*) of the variation in the vegetation variables (Table S4). Climate change was predicted to have a negative effect on most of the vegetation variables with regard to their impact on the focal species (Table 4). While the models suggested a reduction in coniferous tree species and resource trees, the proportion of beech was predicted to increase. A decrease was also predicted for the abundance of gaps and the density of inner forest edges, which would go along with a reduction in ground vegetation cover and basal-branched trees.

**Table 4:** Current conditions (2010) and predicted variable changes between 2010 and 2050 (ΔV 2050) (mean and standard deviation SD) calculated across all sampling plots (n = 4752).

Variable	Unit	2010		ΔV 2050	
		mean	SD	mean	SD
TEMPS	°C	10.93	2.00	1.15	0.35
TEMPW	°C	−2.41	1.37	1.53	0.22
PRECS	mm	146.93	32.81	−6.08	6.75
PRECW	mm	121.09	50.94	−4.66	12.10
BEE	%	18.67	25.64	10.08	1.62
CHEIGHT4	%	74.86	17.60	−1.23	0.35
GAPINDEX	n/ha	7.98	5.71	−1.21	0.25
CHH	m/ha	911.07	396.10	−89.72	24.30
SHRUBCOV	%	15.42	17.49	0.20	0.76
GVCOV	%	54.27	30.28	−14.55	2.39
SPR	%	48.33	33.70	−7.89	1.85
PIN	%	6.20	19.10	−5.78	1.03
RES	%	7.59	13.78	−0.61	0.67
HERB	%	17.42	18.79	−9.47	1.53
FERN	%	4.38	8.85	0.47	0.38
VAC	%	11.07	18.03	−1.96	1.08
STANDDEAD	n/900 m^2^	2.19	4.30	−1.15	0.19
HSTUMP	n/225 m^2^	0.37	1.40	0.12	0.03
ROWANS	n/225 m^2^	1.00	2.95	−0.49	0.18
BBTREE	n/900 m^2^	0.95	2.14	−1.04	0.25
ED134	m/ha	202.06	144.62	−28.15	8.80

Only variables significant in the species’ models are considered. The changes in climate variables were directly obtained from the climate data (model: ECHAM5/CLM, scenario: A1B). Potential vegetation changes were derived from multiple regression models describing vegetation variables as a function of climate (see Table S4) which were calibrated under current (2010) and extrapolated to future (2050) climate conditions.

### Predicted Changes in Habitat Suitability

Climate change was predicted to negatively affect the probability of occurrence of all model species (Table 5). When considering only climatic changes, the greatest impact was predicted for hazel grouse, amounting to a reduction of presence probability of 29% in the currently occupied sites. The least-affected was the three-toed woodpecker with −22%, while capercaillie (−27%) and pygmy owl (−24%) were in intermediate positions (Table 5). Yet, when also considering climate-related vegetation changes, a significant additional reduction of presence probability of 14% was recorded for capercaillie while the conditions for the other three species remained more or less constant. Predicted climate change effects differed greatly between the four study regions, with the greatest impacts on all species recorded for the Black Forest. The Central Eastern Alps were the least affected by climate-change related habitat alterations, except for capercaillie, which was predicted to suffer least in the Swiss Jura and the Northern Prealps (Table S5).

**Table 5:** Modelled probability of species presence (P_pres_) across the study area, as well as mean predicted changes (ΔP_pres_) between 2010 and 2050 under climate change.

Species	2010		Change 2050C		Change 2050CV	
	P_(pres)_	SD	ΔP_(pres)_	SD	ΔP_(pres)_	SD
CC	0.803	0.203	−0.265	0.148	−0.407	0.187
HG	0.795	0.220	−0.292	0.204	−0.302	0.208
TTW	0.717	0.201	−0.222	0.123	−0.215	0.129
PO	0.817	0.226	−0.237	0.333	−0.256	0.346

The first model considers only changes in climate (2050C), the second (2050CV) takes also predicted vegetation changes into account. (CC: capercaillie, HG: hazel grouse, TTW: three-toed woodpecker, PO: pygmy owl).

### Compensation Potential

**Figure 3:**
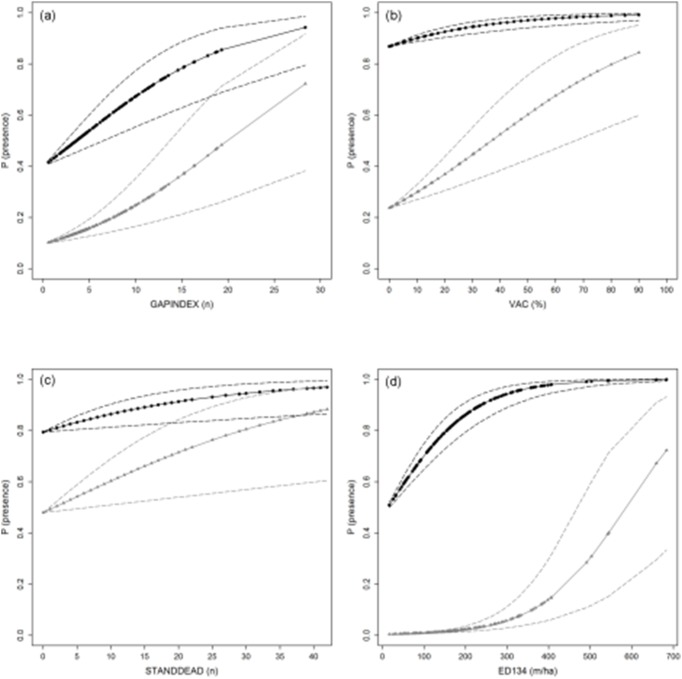
Predicted probability of species presence for (a) capercaillie, (b) hazel grouse, (c) three-toed woodpecker and (d) pygmy owl under current (2010, black) and future (2050, grey) climate conditions, modelled in dependence of the vegetation variable with the highest compensation potential, while holding all other variables constant at their empirical average. Dashed lines indicate the 95% confidence interval. Variable codes are given in Table 2, response curves for all relevant vegetation variables are provided in Figure S2–S5.

**Figure 4:**
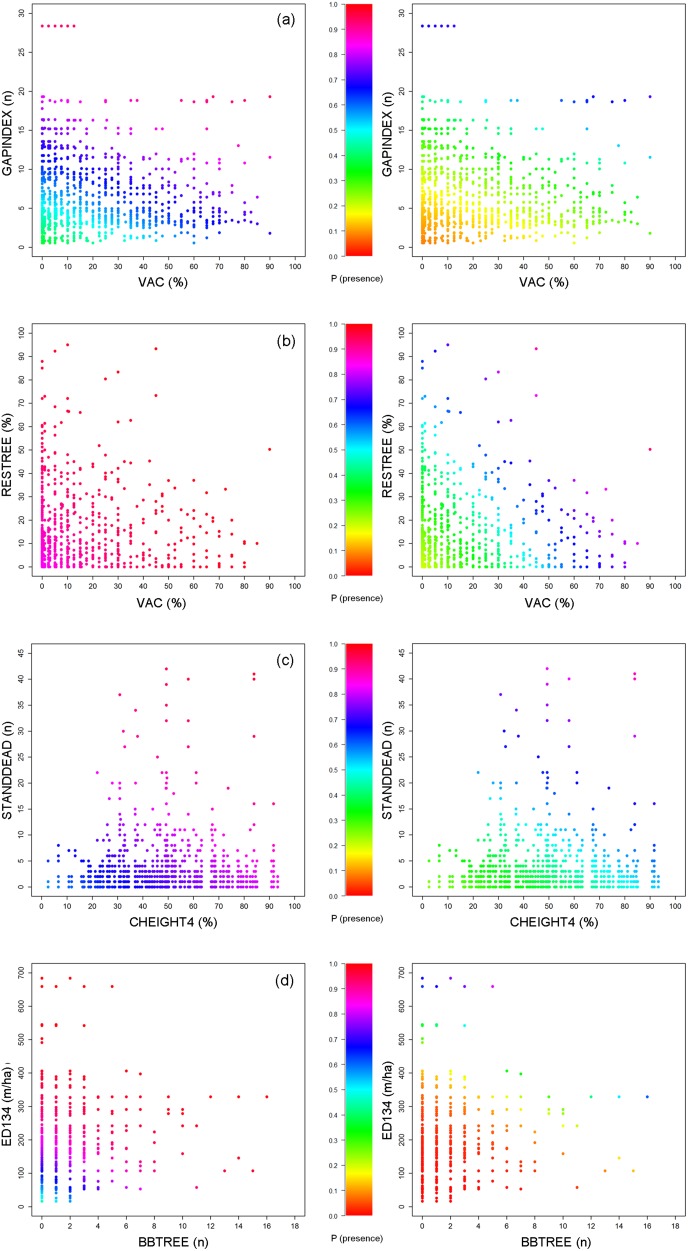
Compensating for climate change effects by modifying in concert the two most upper-ranked vegetation variables per species: predicted probability of species presence (colour scale) for (a) capercaillie, (b) hazel grouse, (c) three-toed woodpecker and (d) pygmy owl under current (2010, left) and future (2050, right) climate conditions, modeled in dependence of the two vegetation variables with the highest compensation potential, while holding all other variables constant at their empirical average. For variable codes see Table 2.

The mean compensation potential, defined as the maximum increase in presence probability (ΔP_(presence)_) under the selected scenario of climate change, ranged between 0.02 (95% confidence interval CI: 0–0.05) for ground vegetation cover (GVCOV) and 0.72 (0.33–0-93) for the density of sharp edges (ED134), both in pygmy owl (Table 6). Adverse effects of climate change on capercaillie could be compensated best by increasing the number of gaps (GAPINDEX) from zero to 28 per km^2^, while hazel grouse availed most of an increase in bilberry (VAC) and resource trees (RESTREE). Increasing the number of snags (STANDDEAD) and the proportion of canopy heights >15 m (CHEIGHT4) most benefitted the tree-toed woodpecker. However, the comparison of the target species’ response curves under current and future climate conditions also showed that it was difficult or even impossible to maintain the prevailing occurrence probability by modifying only one vegetation variable (Figure 3, Figure S2). A considerable increase could be achieved by changing more than one variable towards the species’ optimum (Figure 4): For capercaillie a ΔP_(presence)_ of 0.65 could be achieved when both VAC and GAPINDEX were modified so as to reach their optimal values. The maximum compensation potential for hazel grouse reached 0.73 with optimal proportions of RESTREE and VAC, while combining a maximum ED134 with a high number of basal-branched trees (BBTREE) increased ΔP_(presence)_ for pygmy owl to 0.82. The probability of three-toed woodpecker presence could be increased by 0.65 when changing CHEIGHT4 and STANDDEAD towards their recorded maximum and by 0.77 when the latter variable was in combination with no recent harvesting activities.

**Table 6:** Compensation potential, defined as the maximally achievable increase in predicted probability of species presence ΔP_(pres)_ under altered climate conditions, which could be obtained when modifying the respective variable from its recorded minimum (Min) towards the species’ optimum (Opt).

Variable (unit)	Min ->Opt.	CC	HG	TTW	PO
		ΔP_(pres)_	ΔP_(pres)_	ΔP_(pres)_	ΔP_(pres)_
	0 ->100				
CHEIGHT4 (%)	(0 ->70<-100)*	0.22 (0.08–0.43)		**0.30 (0.12–0.47)**	0.09 (0.02–0.28)
GAPINDEX (n)	0 ->28	**0.62 (0.28–0.82)**			
ED134 (m/ha)	0 ->700				**0.72 (0.33–0.93**)
GVCOV (%)	0 ->100				0.02 (0.00–0.05)
SPR (%)	0 ->70<-100			0.37 (0.25–0.50)	
PIN (%)	0 ->100			0.35 (0.11–0.46)	
RESTREE (%)	0 ->100		**0.48 (0.10–0.68)**	0.29 (0.04–0.43)	
HERB (%)	0 ->100		0.31 (0.09–0.52)		
VAC (%)	0 ->100	**0.27 (0.06–0.50)**	**0.61 (0.36–0.71)**		
BBTREE (n)	0 ->18		0.44 (0.04–0.66)		**0.29 (0.03–0.78)**
STANDDEAD (n)	0 ->42			**0.40 (0.12–0.49)**	
HSTUMP (n)	16 ->0	0.19 (0.10–0.20)		0.41 (0.15–0.50)	

Mean and 95% confidence interval are provided. The two variables that were modified in concert to show their combined compensation potential (Figure 4) are highlighted in bold. (CC: capercaillie, HG: hazel grouse, TTW: three-toed woodpecker, PO: pygmy owl).

*for capercaillie.

## Discussion

Despite widespread calls for adapting biodiversity conservation to the predicted impacts of climate change, the majority of strategies described in the literature are general recommendations that do not specify how to implement them in a real-world context [72]. This may partially be due to the high level of uncertainty in predicting the effects of climate-change on species and habitats [73] and the consequential ecological and economic risks associated with investing in an uncertain outcome. Our study is an attempt to reinforce the general principal of “securing populations by intensive management” (Heller and Zavaleta 2009) by evaluating the general potential and specific measures for compensating adverse effects of climate change on selected forest bird species. Nevertheless, within the presented framework, our study is subjected to the same limitations and sources of uncertainty inherent in predictive species-habitat modelling: First, our models are correlative, and based on the premise of niche conservatism [74]–[76], i.e. they assume that contemporary species-habitat associations will remain unchanged under altered climate conditions and that future changes in biotic interactions such as interspecific competition or predator-prey relationships will not modify them in essence. Second, our outcome may be subject to a considerable level of uncertainty that can arise from variation in input data [54], [55], statistical methods [55], [56] or model parameterization [57], [58]. Since these aspects have been evaluated earlier [59], we deliberately restricted our analyses to one climate change scenario, one global and regional circulation model and one statistical modelling approach, inferring that methodological variations may have changed the absolute values but not the general direction of our outcomes.

### Contemporary Species Habitat Relations

Forest biodiversity will substantially be influenced by the interactions of climate change, forest management and the response of individual species. Our species data stemmed from different regions, covering a broad gradient of climate conditions, landscape and land use characteristics as well as forest management regimes, representative for the model species’ Central European habitats and chosen so as to maximize the generality of the results. The relevant habitat variables identified for the four model species were thus largely in line with the findings of earlier studies. While there was a convergent selection pattern regarding climate and most landscape characteristics, different variables pertaining to vegetation structure and composition were important to the different species, supporting their hypothesized complementarity regarding the indicator function for forest structural attributes [19], [20]. Surprisingly, climate variables significantly contributed to explaining the occurrence of all species, despite the fact that our sampling strategy confined the selection of absence cells to the altitudinal-climatic range that could potentially be occupied by the species. This suggests that - in addition to climate-related vegetation composition and structure - climatic conditions *per se*, or climate-related impacts not captured in this study (e.g., interspecific competition, predator-prey relationships or parasite abundance), play a role in the species’ regional distribution pattern.

### Climate Effects on Vegetation Characteristics

All vegetation variables were significantly correlated with climatic predictors, yet, the proportion of explained variance was very low in some variables. The strongest correlations were found for the relative abundance of tree species such as *Fagus sylvatica* and *Pinus spp,* as well as for ground vegetation cover which reflects sub-canopy light conditions and thus also serves as an indicator for canopy density. Whereas the competitiveness of tree species is directly determined by climatic conditions, differences in canopy and ground vegetation cover may be attributed to longer vegetation periods and accelerated tree-growth in the lower altitudes, and to a higher abundance of beech-dominated forests with naturally scarcer ground vegetation. Yet, although climate is an important driver for shaping vegetation patterns, some of the correlations may be spurious. Hard stumps indicating harvesting activities, or the abundance of snags might be more affected by site-accessibility and topographically divergent patterns of forest management than by climate [77].

The observed correlations support the indirect effect of climate on the model species showing that favored climatic conditions mostly go along with favorable vegetation conditions. They also suggest expected vegetation changes under altered climate conditions to be mostly to the disadvantage of the species. Consequently, not only vegetation-related habitat suitability for our model species is expected to decrease with climate change; also the effort necessary to maintain or improve vegetation characteristics of suitable forest stands will increase because habitat management will have to additionally compensate for adverse natural dynamics. Of course, our simplified approach neglects site conditions, past management regimes and disturbance events and can thus only indicate rough vegetation trends. This may be a reason why the predicted decline of hazel grouse occurrence might be too large. The species is considered to profit from more frequent natural disturbances (e.g., wind storms) under future climatic conditions [78]. Thus, more sophisticated models that incorporate the occurrence of natural disturbances would be desirable and increase the accuracy of models in the future.

### Effects of Climate Change on Habitat Suitability

Occurrence probability, as a proxy for habitat suitability, was predicted to decrease with climate change in all four species, although the magnitude of change differed between species and regions. As expected, impacts were greatest in the Black Forest located at the edge of the species’ bioclimatic envelope [9], while habitats in the Central Eastern Alps were least affected - except for capercaillie, which seems to be the most sensitive and specialized among the four species [59]. Reduction in occurrence probability was mostly caused by changes in climate parameters. A further decrease was observed when additionally including climate-induced vegetation changes, although this was only significant for capercaillie. However, the relative importance of correlated predictors or predictor sets remains difficult to assess in a correlative modelling framework [79]. For disentangling climatic and climate-induced vegetation effects a causality-based, experimental approach may be required.

Although our predictions refer to 2050, a time-lag in the actual response can be expected due to the low reactivity, long generation times and low natural migration rates of tree species in a forest ecosystem [80]. Although, due to their high mobility, bird species are generally attributed a high capacity to track environmental changes, our model species show specific traits which might increase this time-lag even further. All four species are non-migratory, characterized by a high degree of specialization, longevity and site fidelity [81], [82], attributes that may impede rapid responses to habitat alterations [83]. The grouse species are additionally characterized by limited dispersal abilities which inhibits exchanges between the mountain ranges [84]–[86] and underlines the necessity for on-site conservation efforts if populations should be maintained.

### Compensation Potential and Adaptive Conservation Management

Recommendations about how to define desired target states and suitable adaptation strategies for biodiversity conservation under climate change cover a wide range of different approaches. Heller and Zavaleta [74] subsumed them to three main strategies: anticipatory reserve selection to secure future hotspots of biodiversity, improvement of landscape connectivity to allow species to track climate change, and on-site management to either increase the resilience or the resistance of populations or ecosystems to climate change. Although the possibility to preserve climate vulnerable species in their current habitats is frequently questioned [52], [53], we show that intensive management, enhancing the species-relevant vegetation structures, offers the potential to compensate for adverse effects of climate change. Yet, for our study species, a full compensation was difficult to achieve and mostly required the modification of more than one habitat feature. Moreover, the “optimal habitat values”, i.e. the values that would be required to achieve maximum compensation, can rarely be reached under real-world conditions. Especially in count-variables, such as the abundance of snags (for three-toed woodpecker), basal-branched trees (for pygmy owl) or gaps (for capercaillie) the optimum (corresponding to the maximum), is largely determined by locally extremely high variable values. While patches with the observed maximum of 42 snags per 900 m^2^ (dbh>12 cm) may be locally beneficial for the three-toed woodpecker, these values must certainly not be achieved across the whole area [42]. Consequently, for defining reasonable target values for management, the spatial distribution and average abundance of key variables across the whole potential habitat area (sensu [16]) must be taken into consideration.

Furthermore, some variables are characterized by a trade-off between feature abundance and size: a high number of 28 gaps (<0.1 ha) per hectare may be possible if they are small, whereas fewer but larger gaps may also be sufficient for capercaillie. Suchant and Braunisch [87], [88] recommend a minimum of 10% of the forest area to be gaps, which might be a more appropriate prescription.

While most measures increasing structural diversity can readily be implemented, changes in forest composition require considerable time and effort. Converting the tree species portfolio towards more drought-adapted tree species is the most important strategy for maintaining forest productivity under climate change, particularly in regions with a pronounced legacy of former silviculture that promoted drought-intolerant tree species, e.g. spruce, outside their natural range. Whether this conversion will support or be in conflict with the habitat requirements of our target species will strongly depend on the selected tree-species: whereas replacing non site-adapted spruce by fir or pine may maintain or even enhance habitat suitability, a promotion of beech would contribute to habitat deterioration of capercaillie and hazel grouse as it would have a direct impact on the ground vegetation. This is particularly to be expected in the Black Forest habitats and at the lower edge of their distribution range.

To tap the full compensation potential, the combined effect of moderately modifying different variables has to be considered and measures must be flexibly adapted to the local site conditions. While gaps or edges may be created in all situations, the abundance of bilberry, for example, can only be increased under suitable soil and light conditions. Yet, despite the high efforts and inherent limitations, habitat improvement may be the favorable option in an uncertain future: simulation studies showed that habitat improvement led to higher species survival under climate change than creating new habitats in prospectively suitable locations [89]. Considering the high divergence between species range forecasts under climate change, enhancing structural complexity in currently occupied habitats represents a conservative “no-regret” strategy - particularly in forest ecosystems which are well known for their moderating effect on local climate conditions compared to open habitats [90].

## Conclusions

Our study shows that intensive habitat management focusing on a relatively small set of decisive variables can buffer indirect negative effects of climate change on forest-dwelling species although it partly requires working against the natural dynamics. This raises several questions in a system of multifunctional forestry where adaptive conservation management has to be balanced with other ecosystem services [91]. First, which target values can be maximally and realistically achieved? Second, where to prioritize investments? And finally, how can long-term implementation be guaranteed in an ecologically and economically sustainable way?

The first aspect is mainly subject to societal values and political decisions [14]. Compensation measures can be costly and in conflict with economic goals and adaptive strategies to manage renewable resources under climate change. The long-term success of the measures taken will therefore depend on public acceptance and a cost-effective planning and integration in regular forest management. To achieve this, investments should be prioritized in areas large enough to support minimum viable populations of the target species, with a key function for population connectivity or functionality. Moreover, areas should be preferred where – based on the prevailing site- and stocking conditions – the expected climate-change impacts can be compensated with a justifiable management effort. Finally, enhancing structural diversity will not solely increase the model species’ resistance towards climate change. Diverse forests with mixed stands, providing multiple niches for both native and immigrating species, are considered a major prerequisite for ecosystem resilience [92]. Measures aiming at preserving indicators of structural diversity may therefore be beneficial to a wide range of taxa of the representative species community, even when the target species may decline or finally go extinct.

## Supporting Information

Figure S1 **Distribution of species data.** 1 km grid cells with presence (white) and absence (black) of (a) capercaillie, (b) hazel grouse, (c) three-toed woodpecker and (d) pygmy owl in the four study regions Black Forest (BF), Swiss Jura (J), Northern and Prealps (NPA), and Central Alps (CEA).
(PDF)

Figure S2 **Predicted probability of capercaillie presence for current (2010, black) and future (2050, grey) climate conditions (a–d).** Presence probability was modeled in dependence of species-relevant vegetation variables, while holding all other variables at their empirical sampling average. For variable codes see Table 2.
(PDF)

Figure S3 **Predicted probability of hazel grouse presence for under current (2010, black) and future (2050, grey) climate conditions (a–d).** Presence probability was modeled in dependence of species-relevant vegetation variables, while holding all other variables at their empirical sampling average. For variable codes see Table 2.
(PDF)

Figure S4 **Predicted probability of three-toed woodpecker presence for under current (2010, black) and future (2050, grey) climate conditions (a–f).** Presence probability was modeled in dependence of species-relevant vegetation variables, while holding all other variables at their empirical sampling average. For variable codes see Table 2.
(PDF)

Figure S5 **Predicted probability of pygmy owl presence for under current (2010, black) and future (2050, grey) climate conditions (a–d).** Presence probability was modeled in dependence of species-relevant vegetation variables, while holding all other variables at their empirical sampling average. For variable codes see Table 2.
(PDF)

Table S1 **Study locations in the four study regions Black Forest (BF), Swiss Jura (J), Northern Prealps (NPA) and Central Eastern Alps (CEA).** Grid cells (1 km^2^) are represented by their centroid, with the location given in DHDN/3-degree Gauss-Kruger zone 3 (GAUSS) and in the Swiss coordinate system CH1903 (SG). Grid cells entirely or partly located within protected areas without public access and the authority that issued the permit for vegetation mapping are indicated.
(PDF)

Table S2 **Accuracy of the models for capercaillie (CC), hazel grouse (HG), three-toed woodpecker (TTW) and pygmy owl (PO).** Model fit is indicated by sensitivity, specificity, the percent correctly classified (PCC) and Cohen’s Kappa (κ_max) at its optimal threshold, as well as the area under the receiver operating characteristics curve (AUC).
(PDF)

Table S3 **Final models for (a) Capercaillie, (b) Hazel grouse, (c) Three-toed woodpecker and (d) Pygmy owl.** The codes for retained variables of the main variable categories C = climate, L = landscape and V = vegetation are provided in Table 2. The variables that were tested for their compensation potential (i.e. that could be modified by forest management so as to increase the probability of species presence under climate change) are indicated by asterisks. For variable codes see Table 2.
(PDF)

Table S4 **Multiple linear regression models describing the correlation of vegetation variables selected in the species models as a function of climate variables.** Models were calculated across all sampling plots in the study area. For variable codes see Table 2.
(PDF)

Table S5 **Modelled probability of species presence (P_pres_) at the presence plots in the four study regions (Black Forest BF, Swiss Jura J, Northern Prealps NPA and Central Eastern Alps CEA), as well as mean predicted changes thereof (ΔP_pres_) between 2010 and 2050 under climate change.** The first model considers only changes in climate variables (2050C), the second (2050CV) additionally takes predicted vegetation changes into account. CC: Capercaillie, HG: Hazel grouse, TTW: Three-toed woodpecker, PO: Pygmy owl.
(PDF)

Appendix S1 **Sources of the geo-data used in this study.** The listing corresponds to the superscripts provided in Table 2.
(PDF)
