# Supplementary material for: Temperate Mountain Forest Biodiversity under Climate Change: Compensating Negative Effects by Increasing Structural Complexity
Source: PLoS One. 2014 May 13;9(5):e97718. doi: 10.1371/journal.pone.0097718 (PMC4019656; doi:10.1371/journal.pone.0097718)

Figure S1: Distribution of 1km grid cells with presence (white) and absence (black) of (a) capercaillie, (b) hazel grouse, (c) three-toed woodpecker and (d) pygmy owl in the four study regions Black Forest (BF), Swiss Jura (J), Northern and Prealps (NPA), and Central Alps (CEA). Geodata: Switzerland: © Bundesamt für Landestopografie Swisstopo (Art. 30 GeoIV): License No.: 5704 000 000, http://www.swisstopo.admin.ch; Germany: © Landesamt für Geoinformation und Landentwicklung Baden-Württemberg, LGL, License No.: 2851.9-1/19, www.lgl-bw.de.

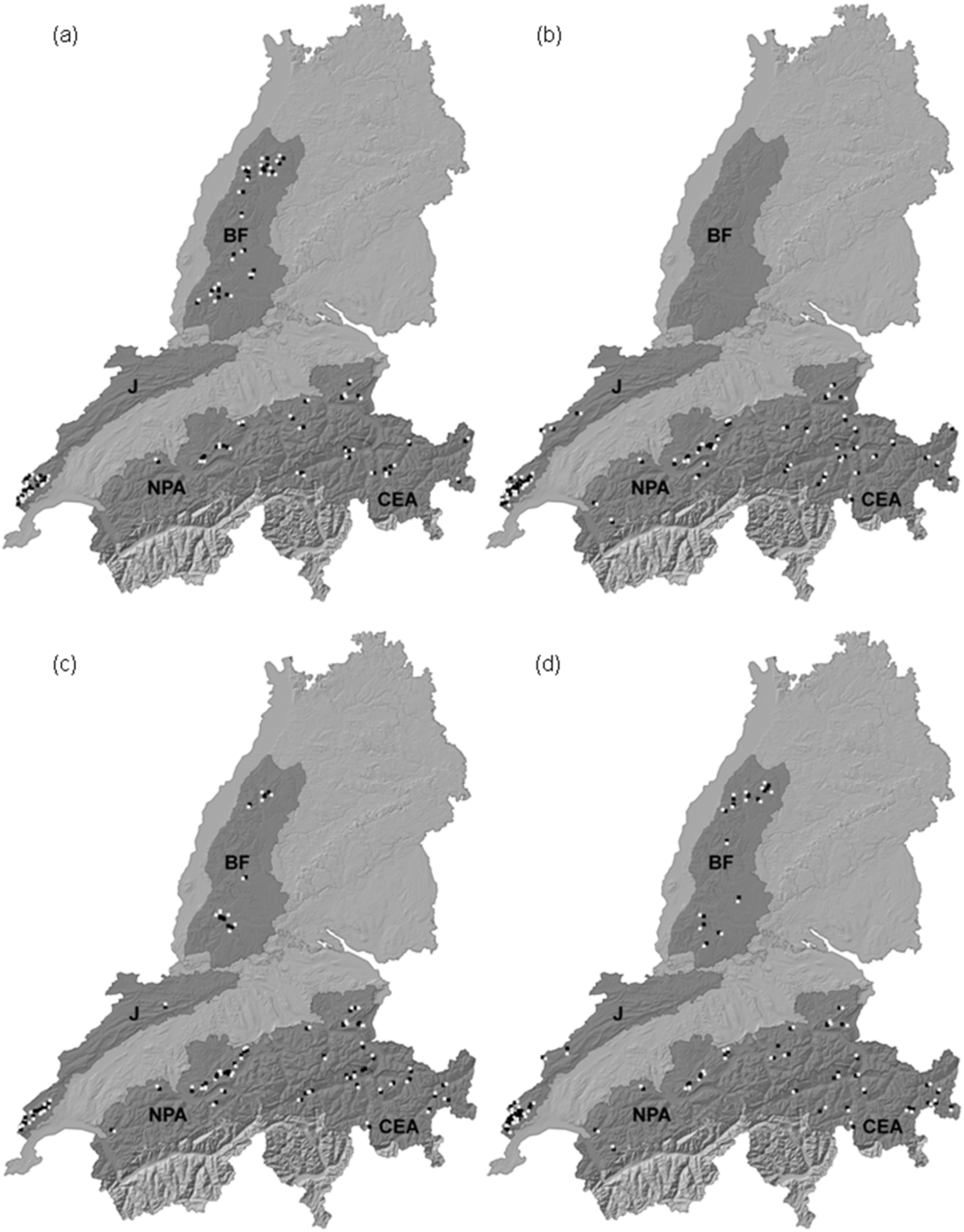

Supplement: Figure S1 — Distribution of species data. 1 km grid cells with presence (white) and absence (black) of (a) capercaillie, (b) hazel grouse, (c) three-toed woodpecker and (d) pygmy owl in the four study regions Black Forest (BF), Swiss Jura (J), Northern and Prealps (NPA), and Central Alps (CEA). (PDF) [file pone.0097718.s001.pdf]
