# Supplementary material for: Temperate Mountain Forest Biodiversity under Climate Change: Compensating Negative Effects by Increasing Structural Complexity
Source: PLoS One. 2014 May 13;9(5):e97718. doi: 10.1371/journal.pone.0097718 (PMC4019656; doi:10.1371/journal.pone.0097718)

Figure S5 a-d: Predicted probability of pygmy owl presence for under current (2010, black) and future (2050, grey) climate conditions, modeled in dependence of species-relevant vegetation variables, while holding all other variables at their empirical sampling average. For variable codes see Table 2.

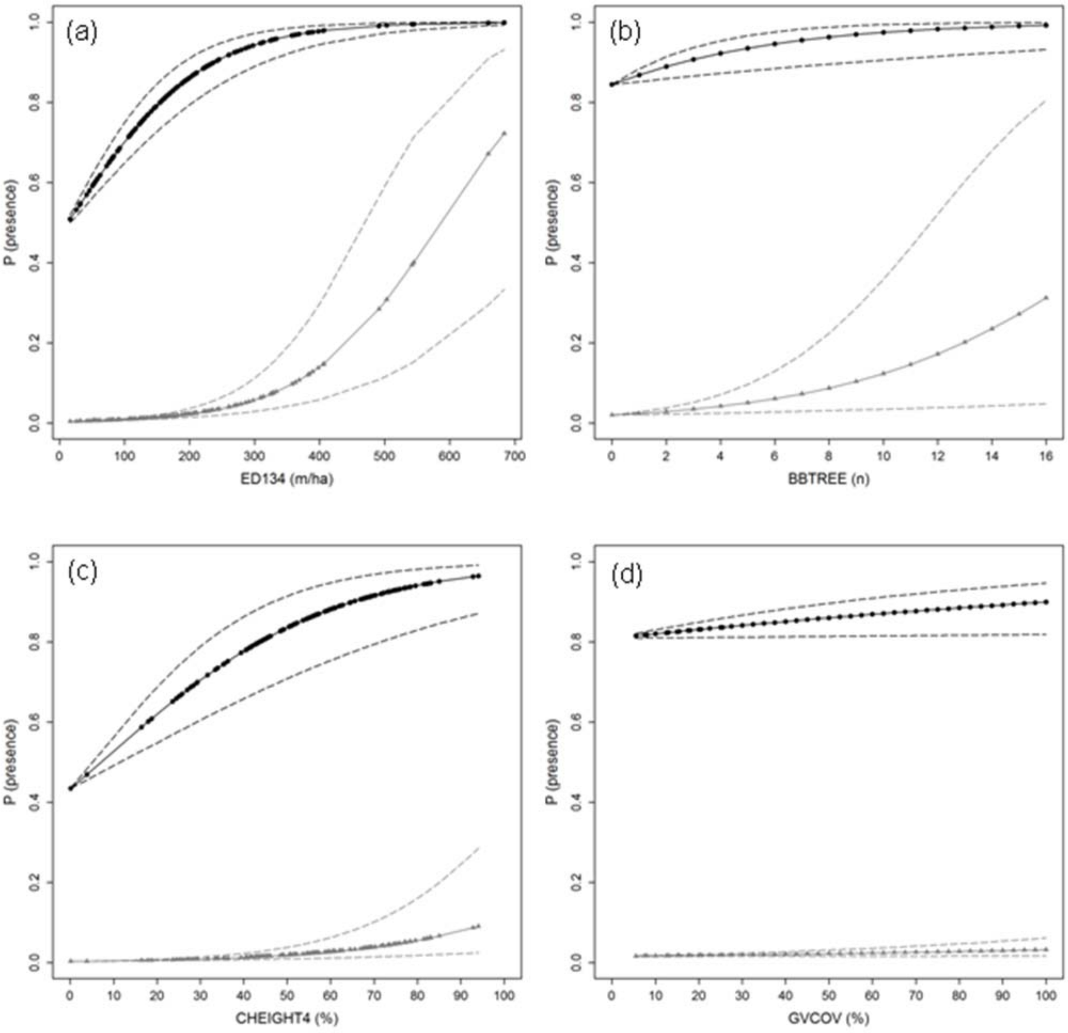

Supplement: Figure S5 — Predicted probability of pygmy owl presence for under current (2010, black) and future (2050, grey) climate conditions (a–d). Presence probability was modeled in dependence of species-relevant vegetation variables, while holding all other variables at their empirical sampling average. For variable codes see Table 2. (PDF) [file pone.0097718.s005.pdf]
