# Supplementary material for: Temperate Mountain Forest Biodiversity under Climate Change: Compensating Negative Effects by Increasing Structural Complexity
Source: PLoS One. 2014 May 13;9(5):e97718. doi: 10.1371/journal.pone.0097718 (PMC4019656; doi:10.1371/journal.pone.0097718)
Supplement: Table S1 — Study locations in the four study regions Black Forest (BF), Swiss Jura (J), Northern Prealps (NPA) and Central Eastern Alps (CEA). Grid cells (1 km2) are represented by their centroid, with the location given in DHDN/3-degree Gauss-Kruger zone 3 (GAUSS) and in the Swiss coordinate system CH1903 (SG). Grid cells entirely or partly located within protected areas without public access and the authority that issued the permit for vegetation mapping are indicated. (PDF) [file pone.0097718.s006.pdf]

Table S1: Study locations in the four study regions Black Forest (BF), Swiss Jura (J), Northern Prealps (NPA) and Central Eastern Alps (CEA). Grid cells ($1km^2$) are represented by their centroid, with the location given in DHDN / 3-degree Gauss-Kruger zone 3 (GAUSS) and in the Swiss coordinate system CH1903 (SG). Grid cells entirely or partly located within protected areas without public access and the authority that issued the permit for vegetation mapping are indicated.

GRID_ID	X_GAUSS	Y_GAUSS	X_SG	Y_SG	Region	Country	Authority
1101	3427000	5286000	644099	284337	BF	Germany	Regional Council Freiburg
1201	3425000	5284000	642140	282297	BF	Germany	NA
1102	3409000	5296000	625902	293974	BF	Germany	Regional Council Freiburg
1202	3410000	5298000	626861	295994	BF	Germany	NA
1103	3434000	5298000	650858	296476	BF	Germany	NA
1203	3436000	5300000	652817	298515	BF	Germany	NA
1104	3431000	5299000	647838	297415	BF	Germany	NA
1204	3432000	5295000	648918	293436	BF	Germany	NA
1105	3425000	5300000	641819	298295	BF	Germany	Regional Council Freiburg
1205	3422000	5297000	638880	295235	BF	Germany	Regional Council Freiburg
1106	3431000	5302000	647778	300415	BF	Germany	Regional Council Freiburg
1206	3434000	5301000	650797	299475	BF	Germany	NA
1108	3428000	5304000	644738	302354	BF	Germany	Regional Council Freiburg
1208	3431000	5307000	647677	305414	BF	Germany	Regional Council Freiburg
1110	3427000	5305000	643718	303334	BF	Germany	Regional Council Freiburg
1210	3425000	5308000	641658	306294	BF	Germany	NA
1111	3425000	5305000	641718	303294	BF	Germany	Regional Council Freiburg
1211	3425000	5310000	641618	308293	BF	Germany	NA
1112	3420000	5305000	636719	303193	BF	Germany	NA
1212	3419000	5302000	635780	300174	BF	Germany	NA
1113	3425000	5306000	641698	304294	BF	Germany	Regional Council Freiburg
1213	3422000	5307000	638679	305233	BF	Germany	Regional Council Freiburg
1114	3449000	5315000	665515	313775	BF	Germany	NA
1214	3448000	5317000	664475	315755	BF	Germany	NA
1115	3451000	5320000	667415	318815	BF	Germany	NA
1215	3450000	5317000	666475	315795	BF	Germany	NA
1116	3436000	5332000	652175	330513	BF	Germany	Regional Council Freiburg
1216	3435000	5328000	651255	326493	BF	Germany	NA
1117	3444000	5335000	660114	333674	BF	Germany	NA
1217	3442000	5334000	658134	332633	BF	Germany	NA
1120	3442000	5362000	657571	360634	BF	Germany	NA
1220	3442000	5360000	657611	358634	BF	Germany	NA
1124	3443000	5378000	658249	376656	BF	Germany	Regional Council Freiburg
1224	3440000	5378000	655248	376596	BF	Germany	NA
1125	3441000	5384000	656127	382617	BF	Germany	NA
1225	3439000	5385000	654107	383577	BF	Germany	NA
1126	3436000	5294000	652938	292516	BF	Germany	NA
1226	3438000	5293000	654957	291556	BF	Germany	NA
1127	3447000	5389000	662028	387739	BF	Germany	NA
1227	3446000	5387000	661068	385718	BF	Germany	NA
1128	3465000	5393000	679951	392102	BF	Germany	NA
1228	3467000	5391000	681992	390141	BF	Germany	NA

1129	3447000	5393000	661947	391740	BF	Germany	NA
1229	3447000	5396000	661886	394740	BF	Germany	NA
1130	3459000	5394000	673930	392981	BF	Germany	NA
1230	3462000	5391000	676991	390041	BF	Germany	NA
1131	3445000	5394000	659926	392699	BF	Germany	NA
1231	3443000	5396000	657885	394659	BF	Germany	NA
1132	3458000	5395000	672909	393962	BF	Germany	Regional Council Freiburg
1232	3456000	5392000	670969	390921	BF	Germany	NA
1133	3458000	5396000	672889	394962	BF	Germany	Regional Council Freiburg
1233	3456000	5398000	670848	396922	BF	Germany	NA
1134	3461000	5398000	675850	397023	BF	Germany	Regional Council Freiburg
1234	3463000	5396000	677890	395062	BF	Germany	NA
1136	3470000	5399000	684832	398204	BF	Germany	Regional Council Freiburg
1236	3468000	5401000	682791	400164	BF	Germany	NA
1137	3472000	5401000	686792	400244	BF	Germany	NA
1237	3475000	5398000	689853	397304	BF	Germany	NA
1138	3473000	5403000	687753	402265	BF	Germany	NA
1238	3470000	5405000	684712	404205	BF	Germany	NA
1139	3461000	5403000	675749	402024	BF	Germany	NA
1239	3457000	5403000	671748	401944	BF	Germany	NA
2101	3276566	5147093	496500	142500	J	Switzerland	NA
2201	3279607	5149035	499500	144500	J	Switzerland	NA
2102	3281922	5165001	501500	160500	J	Switzerland	NA
2202	3282883	5162981	502500	158500	J	Switzerland	NA
2103	3281764	5156998	501500	152500	J	Switzerland	NA
2203	3278744	5156057	498500	151500	J	Switzerland	NA
2104	3289925	5164844	509500	160500	J	Switzerland	NA
2204	3290964	5166825	510500	162500	J	Switzerland	NA
2105	3280744	5156018	500500	151500	J	Switzerland	NA
2205	3284707	5153938	504500	149500	J	Switzerland	NA
2106	3279665	5152036	499500	147500	J	Switzerland	NA
2206	3281666	5151997	501500	147500	J	Switzerland	NA
2107	3296966	5166706	516500	162500	J	Switzerland	NA
2207	3298987	5167667	518500	163500	J	Switzerland	NA
2108	3293906	5163765	513500	159500	J	Switzerland	NA
2208	3296907	5163705	516500	159500	J	Switzerland	NA
2109	3292985	5167785	512500	163500	J	Switzerland	NA
2209	3290945	5165824	510500	161500	J	Switzerland	NA
2110	3290846	5160823	510500	156500	J	Switzerland	NA
2210	3293847	5160764	513500	156500	J	Switzerland	NA
2111	3331926	5214042	550500	210500	J	Switzerland	NA
2211	3334927	5213982	553500	210500	J	Switzerland	NA
2113	3314684	5202378	533500	198500	J	Switzerland	NA
2213	3312704	5203418	531500	199500	J	Switzerland	NA
2114	3282784	5157979	502500	153500	J	Switzerland	NA
2214	3284765	5156939	504500	152500	J	Switzerland	NA
2115	3282942	5165982	502500	161500	J	Switzerland	NA
2215	3285963	5166923	505500	162500	J	Switzerland	NA
2116	3287806	5158881	507500	154500	J	Switzerland	NA
2216	3291807	5158802	511500	154500	J	Switzerland	NA

2117	3283804	5158960	503500	154500	J	Switzerland	NA
2217	3281823	5160000	501500	155500	J	Switzerland	NA
2118	3305662	5201557	524500	197500	J	Switzerland	NA
2218	3307643	5200517	526500	196500	J	Switzerland	NA
2119	3283864	5161961	503500	157500	J	Switzerland	NA
2219	3281843	5161000	501500	156500	J	Switzerland	NA
2120	3276645	5151094	496500	146500	J	Switzerland	NA
2220	3280646	5151016	500500	146500	J	Switzerland	NA
2121	3288846	5160862	508500	156500	J	Switzerland	NA
2221	3287924	5164883	507500	160500	J	Switzerland	NA
2122	3284982	5167943	504500	163500	J	Switzerland	NA
2222	3287003	5168904	506500	164500	J	Switzerland	NA
2123	3287865	5161882	507500	157500	J	Switzerland	NA
2223	3284844	5160941	504500	156500	J	Switzerland	NA
2124	3284825	5159941	504500	155500	J	Switzerland	NA
2224	3282844	5160980	502500	156500	J	Switzerland	NA
2125	3287747	5155880	507500	151500	J	Switzerland	NA
2225	3286707	5153899	506500	149500	J	Switzerland	NA
2126	3282725	5154978	502500	150500	J	Switzerland	NA
2226	3284687	5152938	504500	148500	J	Switzerland	NA
2127	3323805	5208200	542500	204500	J	Switzerland	NA
2227	3323726	5204200	542500	200500	J	Switzerland	NA
2128	3278606	5149055	498500	144500	J	Switzerland	NA
2228	3279646	5151036	499500	146500	J	Switzerland	NA
2129	3279587	5148035	499500	143500	J	Switzerland	NA
2229	3281548	5145995	501500	141500	J	Switzerland	NA
2130	3290807	5158822	510500	154500	J	Switzerland	NA
2230	3292788	5157782	512500	153500	J	Switzerland	NA
2131	3295946	5165726	515500	161500	J	Switzerland	NA
2231	3297927	5164686	517500	160500	J	Switzerland	NA
2132	3295927	5164725	515500	160500	J	Switzerland	NA
2232	3294867	5161744	514500	157500	J	Switzerland	NA
2133	3291886	5162804	511500	158500	J	Switzerland	NA
2233	3292847	5160783	512500	156500	J	Switzerland	NA
2134	3289866	5161843	509500	157500	J	Switzerland	NA
2234	3291827	5159803	511500	155500	J	Switzerland	NA
2135	3297986	5167687	517500	163500	J	Switzerland	NA
2235	3299026	5169668	518500	165500	J	Switzerland	NA
2136	3286786	5157900	506500	153500	J	Switzerland	NA
2236	3286747	5155900	506500	151500	J	Switzerland	NA
2137	3280764	5157018	500500	152500	J	Switzerland	NA
2237	3280705	5154017	500500	149500	J	Switzerland	NA
2138	3279685	5153036	499500	148500	J	Switzerland	NA
2238	3278645	5151055	498500	146500	J	Switzerland	NA
2139	3277586	5148074	497500	143500	J	Switzerland	NA
2239	3276625	5150094	496500	145500	J	Switzerland	NA
2140	3384448	5239003	602500	236500	J	Switzerland	NA
2240	3384488	5241003	602500	238500	J	Switzerland	NA
2141	3293926	5164765	513500	160500	J	Switzerland	NA
2241	3294966	5166746	514500	162500	J	Switzerland	NA

2142	3281686	5152997	501500	148500	J	Switzerland	NA
2242	3283687	5152958	503500	148500	J	Switzerland	NA
2143	3282706	5153978	502500	149500	J	Switzerland	NA
3101	3521424	5236271	739500	236500	NPA	Switzerland	NA
3201	3522464	5238251	740500	238500	NPA	Switzerland	NA
3102	3430506	5191071	649500	189500	NPA	Switzerland	NA
3202	3432466	5189032	651500	187500	NPA	Switzerland	NA
3103	3522404	5235252	740500	235500	NPA	Switzerland	NA
3203	3520384	5234292	738500	234500	NPA	Switzerland	NA
3104	3528244	5227134	746500	227500	NPA	Switzerland	NA
3204	3530203	5225095	748500	225500	NPA	Switzerland	NA
3105	3431486	5190051	650500	188500	NPA	Switzerland	NA
3205	3434486	5189992	653500	188500	NPA	Switzerland	NA
3106	3487774	5203935	706500	203500	NPA	Switzerland	NA
3206	3484755	5202995	703500	202500	NPA	Switzerland	NA
3107	3412290	5180430	631500	178500	NPA	Switzerland	NA
3207	3412250	5178430	631500	176500	NPA	Switzerland	NA
3108	3443822	5206810	662500	205500	NPA	Switzerland	NA
3208	3444862	5208790	663500	207500	NPA	Switzerland	NA
3109	3517226	5226353	735500	226500	NPA	Switzerland	NA
3209	3519226	5226313	737500	226500	NPA	Switzerland	NA
3110	3489152	5222911	707500	222500	NPA	Switzerland	NA
3210	3491172	5223871	709500	223500	NPA	Switzerland	NA
3111	3501752	5202656	720500	202500	NPA	Switzerland	NA
3211	3502712	5200637	721500	200500	NPA	Switzerland	NA
3112	3435625	5196971	654500	195500	NPA	Switzerland	NA
3212	3436585	5194951	655500	193500	NPA	Switzerland	NA
3113	3431506	5191051	650500	189500	NPA	Switzerland	NA
3213	3433466	5189012	652500	187500	NPA	Switzerland	NA
3114	3428487	5190111	647500	188500	NPA	Switzerland	NA
3214	3425507	5191171	644500	189500	NPA	Switzerland	NA
3115	3530885	5209078	749500	209500	NPA	Switzerland	NA
3215	3529945	5212097	748500	212500	NPA	Switzerland	NA
3116	3527224	5226154	745500	226500	NPA	Switzerland	NA
3216	3531203	5225075	749500	225500	NPA	Switzerland	NA
3117	3431466	5189052	650500	187500	NPA	Switzerland	NA
3217	3433446	5188012	652500	186500	NPA	Switzerland	NA
3118	3414329	5182390	633500	180500	NPA	Switzerland	NA
3218	3414250	5178391	633500	176500	NPA	Switzerland	NA
3119	3422051	5168233	641500	166500	NPA	Switzerland	NA
3219	3420011	5166273	639500	164500	NPA	Switzerland	NA
3120	3516226	5226373	734500	226500	NPA	Switzerland	NA
3220	3518266	5228333	736500	228500	NPA	Switzerland	NA
3121	3358344	5133481	578500	130500	NPA	Switzerland	NA
3221	3357384	5135501	577500	132500	NPA	Switzerland	NA
3122	3441803	5205850	660500	204500	NPA	Switzerland	NA
3222	3439823	5206890	658500	205500	NPA	Switzerland	NA
3123	3413310	5181410	632500	179500	NPA	Switzerland	NA
3223	3411250	5178450	630500	176500	NPA	Switzerland	NA
3124	3442803	5205830	661500	204500	NPA	Switzerland	NA

3224	3442843	5207830	661500	206500	NPA	Switzerland	NA
3125	3422448	5188231	641500	186500	NPA	Switzerland	NA
3225	3421388	5185251	640500	183500	NPA	Switzerland	NA
3126	3479895	5210093	698500	209500	NPA	Switzerland	NA
3226	3477936	5212132	696500	211500	NPA	Switzerland	NA
3127	3516206	5225373	734500	225500	NPA	Switzerland	NA
3227	3518246	5227333	736500	227500	NPA	Switzerland	NA
3128	3476717	5201154	695500	200500	NPA	Switzerland	NA
3228	3474717	5201194	693500	200500	NPA	Switzerland	NA
3129	3538724	5200920	757500	201500	NPA	Switzerland	NA
3229	3537764	5202940	756500	203500	NPA	Switzerland	NA
3130	3440743	5202870	659500	201500	NPA	Switzerland	NA
3230	3440704	5200871	659500	199500	NPA	Switzerland	NA
3131	3423467	5189211	642500	187500	NPA	Switzerland	NA
3231	3425467	5189171	644500	187500	NPA	Switzerland	NA
3132	3422428	5187231	641500	185500	NPA	Switzerland	NA
3232	3424507	5191190	643500	189500	NPA	Switzerland	NA
3133	3414310	5181390	633500	179500	NPA	Switzerland	NA
3233	3414270	5179391	633500	177500	NPA	Switzerland	NA
3134	3404211	5176589	623500	174500	NPA	Switzerland	NA
3234	3404271	5179589	623500	177500	NPA	Switzerland	NA
3135	3444822	5206790	663500	205500	NPA	Switzerland	NA
3235	3443862	5208810	662500	207500	NPA	Switzerland	NA
3136	3428208	5176113	647500	174500	NPA	Switzerland	NA
3236	3426229	5177153	645500	175500	NPA	Switzerland	NA
3137	3344620	5147757	564500	144500	NPA	Switzerland	NA
3237	3346600	5146718	566500	143500	NPA	Switzerland	NA
3138	3525463	5238191	743500	238500	NPA	Switzerland	NA
3238	3527462	5238152	745500	238500	NPA	Switzerland	NA
3139	3380233	5178066	599500	175500	NPA	Switzerland	NA
3239	3378273	5180105	597500	177500	NPA	Switzerland	NA
4101	3607596	5193548	826500	195500	CEA	Switzerland	NA
4201	3609635	5195508	828500	197500	CEA	Switzerland	NA
4102	3540107	5169886	759500	170500	CEA	Switzerland	NA
4202	3538107	5169926	757500	170500	CEA	Switzerland	NA
4103	3518470	5188321	737500	188500	CEA	Switzerland	NA
4203	3519430	5186301	738500	186500	CEA	Switzerland	NA
4104	3552204	5174646	771500	175500	CEA	Switzerland	NA
4204	3555224	5175586	774500	176500	CEA	Switzerland	NA
4105	3551105	5169667	770500	170500	CEA	Switzerland	NA
4205	3549065	5167707	768500	168500	CEA	Switzerland	NA
4106	3487098	5169942	706500	169500	CEA	Switzerland	NA
4206	3488058	5167922	707500	167500	CEA	Switzerland	NA
4107	3521330	5181263	740500	181500	CEA	Switzerland	NA
4207	3519350	5182302	738500	182500	CEA	Switzerland	NA
4108	3602997	5163630	822500	165500	CEA	Switzerland	NA
4208	3602038	5165650	821500	167500	CEA	Switzerland	NA
4109	3548165	5172726	767500	173500	CEA	Switzerland	NA
4209	3549105	5169707	768500	170500	CEA	Switzerland	NA
4110	3510934	5161466	730500	161500	CEA	Switzerland	NA

4210	3514992	5164386	734500	164500	CEA	Switzerland	NA
4111	3530348	5182083	749500	182500	CEA	Switzerland	NA
4211	3528328	5181123	747500	181500	CEA	Switzerland	NA
4112	3515052	5167385	734500	167500	CEA	Switzerland	NA
4212	3516092	5169365	735500	169500	CEA	Switzerland	NA
4114	3524508	5190201	743500	190500	CEA	Switzerland	NA
4214	3524468	5188202	743500	188500	CEA	Switzerland	NA
4115	3486118	5170961	705500	170500	CEA	Switzerland	NA
4215	3488157	5172921	707500	172500	CEA	Switzerland	NA
4116	3609775	5202508	828500	204500	CEA	Switzerland	NA
4216	3607815	5204547	826500	206500	CEA	Switzerland	NA
4118	3488197	5174921	707500	174500	CEA	Switzerland	NA
4218	3490217	5175881	709500	175500	CEA	Switzerland	NA
4119	3530547	5192081	749500	192500	CEA	Switzerland	NA
4219	3532566	5193041	751500	193500	CEA	Switzerland	NA
4120	3591079	5167869	810500	169500	CEA	Switzerland	NA
4220	3593058	5166829	812500	168500	CEA	Switzerland	Swiss National Park
4121	3544226	5175806	763500	176500	CEA	Switzerland	NA
4221	3543266	5177825	762500	178500	CEA	Switzerland	NA
4122	3493236	5176821	712500	176500	CEA	Switzerland	NA
4222	3491217	5175861	710500	175500	CEA	Switzerland	NA
4123	3598238	5175729	817500	177500	CEA	Switzerland	Swiss National Park
4223	3599277	5177709	818500	179500	CEA	Switzerland	Swiss National Park
4124	3597078	5167749	816500	169500	CEA	Switzerland	Swiss National Park
4224	3596118	5169769	815500	171500	CEA	Switzerland	NA
4125	3608017	5164530	827500	166500	CEA	Switzerland	NA
4225	3607977	5162530	827500	164500	CEA	Switzerland	NA
4126	3577001	5164149	796500	165500	CEA	Switzerland	NA
4226	3574961	5162190	794500	163500	CEA	Switzerland	NA
4127	3530989	5164067	750500	164500	CEA	Switzerland	NA
4227	3531949	5162047	751500	162500	CEA	Switzerland	NA
4128	3579980	5163090	799500	164500	CEA	Switzerland	Swiss National Park
4228	3578940	5161110	798500	162500	CEA	Switzerland	NA
4129	3537466	5187943	756500	188500	CEA	Switzerland	NA
4229	3539485	5188903	758500	189500	CEA	Switzerland	NA
4131	3566541	5191365	785500	192500	CEA	Switzerland	NA
4231	3565581	5193384	784500	194500	CEA	Switzerland	NA
4132	3521429	5186262	740500	186500	CEA	Switzerland	NA
4232	3524448	5187202	743500	187500	CEA	Switzerland	NA
4133	3565421	5185385	784500	186500	CEA	Switzerland	NA
4233	3563382	5183426	782500	184500	CEA	Switzerland	NA
4134	3553363	5182625	772500	183500	CEA	Switzerland	NA
4234	3551344	5181665	770500	182500	CEA	Switzerland	NA
4135	3590338	5180888	809500	182500	CEA	Switzerland	NA
4235	3592358	5181848	811500	183500	CEA	Switzerland	NA
4136	3526329	5181163	745500	181500	CEA	Switzerland	NA
4236	3527289	5179144	746500	179500	CEA	Switzerland	NA
4137	3584179	5173008	803500	174500	CEA	Switzerland	NA
4237	3584219	5175008	803500	176500	CEA	Switzerland	Swiss National Park
4138	3594118	5169809	813500	171500	CEA	Switzerland	Swiss National Park

4238	3591139	5170869	810500	172500	CEA	Switzerland	NA
4139	3485098	5169982	704500	169500	CEA	Switzerland	NA
4239	3483099	5170021	702500	169500	CEA	Switzerland	NA
4140	3535709	5149970	755500	150500	CEA	Switzerland	NA
4240	3533769	5153010	753500	153500	CEA	Switzerland	NA
