# Supplementary material for: Temperate Mountain Forest Biodiversity under Climate Change: Compensating Negative Effects by Increasing Structural Complexity
Source: PLoS One. 2014 May 13;9(5):e97718. doi: 10.1371/journal.pone.0097718 (PMC4019656; doi:10.1371/journal.pone.0097718)
Supplement: Table S2 — Accuracy of the models for capercaillie (CC), hazel grouse (HG), three-toed woodpecker (TTW) and pygmy owl (PO). Model fit is indicated by sensitivity, specificity, the percent correctly classified (PCC) and Cohen’s Kappa (κ_max) at its optimal threshold, as well as the area under the receiver operating characteristics curve (AUC). (PDF) [file pone.0097718.s007.pdf]

Table S2: Accuracy of the models for capercaillie (CC), hazel grouse (HG), three-toed woodpecker (TTW) and pygmy owl (PO). Model fit is indicated by sensitivity, specificity, the percent correctly classified (PCC) and Cohen's Kappa (κ_max) at its optimal threshold, as well as the area under the receiver operating characteristics curve (AUC).

Model	threshold	sensitivity	SD	specificity	SD	PCC	SD	κ_max	SD	AUC	SD
CC	0,520	0,896	0,011	0,780	0,018	0,851	0,010	0,683	0,020	0,931	0,006
HG	0,600	0,833	0,014	0,834	0,017	0,833	0,011	0,659	0,022	0,918	0,008
TTW	0,560	0,802	0,015	0,801	0,017	0,802	0,011	0,601	0,023	0,877	0,010
PO	0,580	0,860	0,012	0,899	0,011	0,878	0,008	0,757	0,016	0,947	0,005
