# Supplementary material for: Temperate Mountain Forest Biodiversity under Climate Change: Compensating Negative Effects by Increasing Structural Complexity
Source: PLoS One. 2014 May 13;9(5):e97718. doi: 10.1371/journal.pone.0097718 (PMC4019656; doi:10.1371/journal.pone.0097718)
Supplement: Table S3 — Final models for (a) Capercaillie, (b) Hazel grouse, (c) Three-toed woodpecker and (d) Pygmy owl. The codes for retained variables of the main variable categories C = climate, L = landscape and V = vegetation are provided in Table 2. The variables that were tested for their compensation potential (i.e. that could be modified by forest management so as to increase the probability of species presence under climate change) are indicated by asterisks. For variable codes see Table 2. (PDF) [file pone.0097718.s008.pdf]

Table S3: Final models for (a) Capercaillie, (b) Hazel grouse, (c) Three-toed woodpecker and (d) Pygmy owl. The codes for retained variables of the main variable categories C=climate, L=landscape and V=vegetation are provided in Table 2. The variables that were tested for their compensation potential (i.e. that could be modified by forest management so as to increase the probability of species presence under climate change) are indicated by asterisks. For variable codes see Table 2.

(a)

Category	Variable	Estimate	SE	P-value
	(Intercept)	-4.370	0.799	***
C	TEMPW	-1.651	0.255	***
	TEMPW ^2	-0.137	0.050	*
	PRECS	0.017	0.004	***
L	EAST	0.353	0.117	**
	SLOPE	-0.009	0.010	n.s.
	WETSOIL	0.040	0.015	**
	FEDGEOUT	-0.242	0.029	***
	FEDGEIN	-0.050	0.023	n.s.
	ROADDENS	-2.73E-04	1.05E-04	***
	SETTLEDIST	1.91E-04	9.26E-05	*
V	*CHEIGHT4	0.094	0.017	***
	CHEIGHT4^2	-8.95E-04	1.63E-04	***
	*GAPINDEX	0.113	0.025	***
	CHH	-2.21E-03	3.75E-04	***
	GVDIS (2: patchy)	0.543	0.331	n.s.
	GVDIS (3: clumped)	-0.043	0.310	n.s.
	*BEE	-0.047	0.010	***
	BEE^2	4.16E-04	1.30E-04	**
	HERB	0.006	0.005	n.s.
	*VAC	0.014	0.005	**
	*HSTUMP	-0.158	0.055	**
	ROW	0.033	0.031	n.s.
	E1	0.529	0.343	n.s.

(b)

Category	Variable	Estimate	SE	P-value
	(Intercept)	-15.980	1.805	***
C	TEMPW	-2.700	0.601	***
	TEMPW ^2	-0.235	0.096	*
	PRECS	0.024	0.009	**
	PRECW	-0.001	0.007	n.s.
L	SOLAR	3.17E-05	7.26E-06	***
	INTENSIVE	-0.057	0.021	**
	ROADDENS	-1.47E-06	1.42E-04	n.s.
	SETTLEDIST	1.43E-04	1.41E-04	n.s.
V	*CHEIGHT4	0.012	0.006	n.s.
	GVDIS (2: patchy)	0.893	0.436	*
	GVDIS (3: clumped)	0.332	0.369	n.s.

	*RESTREE	0.023	0.009	*
	*HERB	0.016	0.005	**
	FERN	0.014	0.010	n.s.
	*VAC	0.034	0.007	***
	ROW	0.037	0.042	n.s.
	*BBTREE	0.104	0.048	*
	E2	-0.640	0.308	*

(c)

Category	Variable	Estimate	SE	P-value
	(Intercept)	-5.396	0.656	***
C	TEMPW	-1.463	0.298	***
	TEMPW ^2	-0.171	0.057	**
	PRECS	0.011	0.003	***
L	SOLAR	0.037	0.010	***
	WETSOIL	-0.145	0.023	***
V	*CHEIGHT4	0.014	0.004	***
	STANDSTRU 2	-0.562	0.194	**
	STANDSTRU 3	-0.608	0.189	**
	SHRUBCOV	-0.012	0.005	*
	*SPR	0.052	0.010	***
	SPR^2	-3,81E-04	9,03E-05	***
	*PIN	0.017	0.006	**
	*RESTREE	0.013	0.006	*
	*STANDDEAD	0.050	0.019	**
	*HSTUMP	-0.131	0.047	**

(d)

Category	Variable	Estimate	SE	P-value
	(Intercept)	-35.120	2.670	***
C	TEMPW	-5.555	0.592	***
	TEMPW ^2	-0.734	0.083	***
	PRECS	0.104	0.022	***
	P122	0.060	0.016	***
L	EAST	0.354	0.141	*
	SLOPE	-0.033	0.012	**
	WETSOIL	0.177	0.028	***
	INTENSIVE	0.074	0.024	**
	ROADDENS	-7.74E-04	1.77E-04	***
	SETTLEDIST	9.27E-04	1.67E-04	***
V	*CHEIGHT4	0.032	0.007	***
	*ED134	0.010	0.001	***
	*GVCOV	0.007	0.003	*
	*BBTREE	0.197	0.069	**
