# Supplementary material for: Temperate Mountain Forest Biodiversity under Climate Change: Compensating Negative Effects by Increasing Structural Complexity
Source: PLoS One. 2014 May 13;9(5):e97718. doi: 10.1371/journal.pone.0097718 (PMC4019656; doi:10.1371/journal.pone.0097718)
Supplement: Table S4 — Multiple linear regression models describing the correlation of vegetation variables selected in the species models as a function of climate variables. Models were calculated across all sampling plots in the study area. For variable codes see Table 2. (PDF) [file pone.0097718.s009.pdf]

Table S4: Multiple linear regression models describing the correlation of vegetation variables selected in the species models as a function of climate variables, calculated across all sampling plots in the study area. For variable codes see Table 2.

Variable	Predictor	Estimate	SE	t	Pr(>|t|)	R^2
BEE	(Intercept)	35.305	2.014	17.534	***	0.188
	PRECS	-0.094	0.015	-6.493	***	
	PRECW	0.093	0.009	10.018	***	
	TEMPW	6.514	0.317	20.539	***	
CHEIGHT4	(Intercept)	81.128	1.306	62.096	***	0.022
	PRECS	-0.044	0.009	-5.077	***	
	PRECW	-0.018	0.006	-3.039	**	
	TEMPW	-1.039	0.200	-5.192	***	
GAPINDEX	(Intercept)	3.317	0.405	8.184	***	0.043
	PRECS	0.020	0.003	8.041	***	
	TEMPW	-0.712	0.061	-11.726	***	
CHH	Intercept)	504.987	28.276	17.859	***	0.096
	PRECS	2.894	0.188	15.364	***	
	PRECW	-1.135	0.128	-8.857	***	
	TEMPW	-50.750	4.331	-11.718	***	
SHRUBCOV	(Intercept)	19.136	1.402	13.652	***	0.029
	PRECS	-0.079	0.011	-7.469	***	
	PRECW	0.061	0.006	9.396	***	
GVCOV	(Intercept)	18.795	2.333	8.057	***	0.179
	PRECS	0.103	0.015	6.77	***	
	TEMPW	-9.131	0.351	-26.018	***	
SPR	(Intercept)	14.705	2.830	5.196	***	0.071
	PRECS	0.202	0.020	9.898	***	
	PRECW	-0.045	0.013	-3.423	***	
	TEMPW	-4.500	0.446	-10.095	***	
PIN	(Intercept)	19.894	1.476	13.479	***	0.212
	PRECS	-0.111	0.011	-10.43	***	
	PRECW	-0.062	0.007	-9.101	***	
	TEMPW	-4.422	0.232	-19.021	***	
RESTREE	(Intercept)	-6.731	1.087	-6.192	***	0.051
	PRECS	0.100	0.007	13.479	***	
HERB	(Intercept)	-0.297	1.084	-0.274		0.176
	PRECW	0.030	0.006	5.024	***	
	TEMPW	-6.115	0.229	-26.689	***	
FERN	(Intercept)	-0.917	0.757	-1.211		0.035
	PRECS	0.059	0.005	10.879	***	
	PRECW	-0.017	0.003	-4.8	***	
	TEMPW	0.492	0.119	4.127	***	
VAC	(Intercept)	-11.375	1.517	-7.498	***	0.066
	PRECS	0.117	0.011	10.697	***	
	PRECW	0.032	0.007	4.538	***	
	TEMPW	-0.721	0.239	-3.017	**	
STANDDEAD	(Intercept)	0.377	0.357	1.056		0.091
	PRECS	0.011	0.003	4.263	***	
	PRECW	-0.012	0.002	-7.02	***	
	TEMPW	-0.743	0.056	-13.209	***	
HSTUMP	(Intercept)	0.900	0.119	7.571	***	0.008
	PRECS	-0.003	0.001	-3.413	***	
	TEMPW	0.065	0.018	3.649	***	

ROW	(Intercept)	-1.999	0.264	-7.575	***	0.049
	PRECS	0.008	0.002	4.387	***	
	PRECW	0.010	0.001	8.177	***	
	TEMPW	-0.261	0.041	-6.391	***	
BBTREE	(Intercept)	-2.524	0.169	-14.978	***	0.185
	PRECS	0.004	0.001	3.432	***	
	PRECW	0.012	0.001	14.796	***	
	TEMPW	-0.627	0.027	-23.622	***	
ED134	(Intercept)	75.593	10.309	7.333	***	0.094
	PRECS	1.044	0.069	15.206	***	
	PRECW	-0.528	0.047	-11.337	***	
	TEMPW	-15.907	1.578	-10.08	***	
