# Supplementary material for: Temperate Mountain Forest Biodiversity under Climate Change: Compensating Negative Effects by Increasing Structural Complexity
Source: PLoS One. 2014 May 13;9(5):e97718. doi: 10.1371/journal.pone.0097718 (PMC4019656; doi:10.1371/journal.pone.0097718)
Supplement: Table S5 — Modelled probability of species presence (Ppres) at the presence plots in the four study regions (Black Forest BF, Swiss Jura J, Northern Prealps NPA and Central Eastern Alps CEA), as well as mean predicted changes thereof (ΔPpres) between 2010 and 2050 under climate change. The first model considers only changes in climate variables (2050C), the second (2050CV) additionally takes predicted vegetation changes into account. CC: Capercaillie, HG: Hazel grouse, TTW: Three-toed woodpecker, PO: Pygmy owl. (PDF) [file pone.0097718.s010.pdf]

Table S5: Modelled probability of species presence (P_{pres}) at the presence plots in the four study regions (Black Forest BF, Swiss Jura J, Northern Prealps NPA and Central Eastern Alps CEA), as well as mean predicted changes thereof (ΔP_{pres}) between 2010 and 2050 under climate change. The first model considers only changes in climate variables (2050C), the second (2050CV) additionally takes predicted vegetation changes into account. CC: Capercaillie, HG: Hazel grouse, TTW: Three-toed woodpecker, PO: Pygmy owl.

**Species**	**Time**	**2010**		**Change 2050C**		**Change 2050CV**	
	**Region**	**P(pres)**	SD	**Δ P(pres)**	SD	**Δ P(pres)**	SD
CC	BF	0.750	0.222	-0.327	0.129	-0.487	0.177
	J	0.871	0.125	-0.212	0.125	-0.352	0.156
	NPA	0.834	0.226	-0.236	0.179	-0.357	0.217
	CEA	0.757	0.210	-0.260	0.142	-0.385	0.159
	ALL	0.803	0.203	-0.265	0.148	-0.407	0.187
HG	BF	-					
	J	0.809	0.210	-0.297	0.193	-0.310	0.195
	NPA	0.847	0.182	-0.297	0.233	-0.306	0.237
	CEA	0.660	0.254	-0.271	0.169	-0.276	0.174
	ALL	0.795	0.220	-0.292	0.204	-0.302	0.208
TTW	BF	0.669	0.199	-0.329	0.079	-0.331	0.082
	J	0.712	0.179	-0.190	0.073	-0.193	0.078
	NPA	0.795	0.176	-0.206	0.123	-0.195	0.133
	CEA	0.611	0.210	-0.164	0.130	-0.149	0.124
	ALL	0.717	0.201	-0.222	0.123	-0.215	0.129
PO	BF	0.845	0.169	-0.413	0.386	-0.426	0.395
	J	0.836	0.216	-0.231	0.299	-0.260	0.315
	NPA	0.853	0.240	-0.189	0.319	-0.213	0.339
	CEA	0.691	0.264	-0.024	0.085	-0.027	0.086
	ALL	0.817	0.226	-0.237	0.333	-0.256	0.346
