# Supplementary material for: Temperate Mountain Forest Biodiversity under Climate Change: Compensating Negative Effects by Increasing Structural Complexity
Source: PLoS One. 2014 May 13;9(5):e97718. doi: 10.1371/journal.pone.0097718 (PMC4019656; doi:10.1371/journal.pone.0097718)
Supplement: Appendix S1 — Sources of the geo-data used in this study. The listing corresponds to the superscripts provided in Table 2. (PDF) [file pone.0097718.s011.pdf]

Appendix S1: Sources of the geo-data used in this study. The listing corresponds to the superscripts provided in Table 2.

a) Federal Institute for Forest, Snow and Landscape Research WSL; available upon request: www.wsl.ch
b) Switzerland: © Bundesamt für Landestopografie Swisstopo (Art. 30 GeoIV): License No.: 5704 000 000, http://www.swisstopo.admin.ch.
c) Germany: © Landesamt für Geoinformation und Landentwicklung Baden-Württemberg, LGL, License No.: 2851.9-1/19, www.lgl-bw.de.
d) VECTOR25 © Bundesamt für Landestopografie Swisstopo, (Art. 30 GeoIV): License No.: 5704 000 000, http://www.swisstopo.admin.ch/internet/swisstopo/-de/home/products.
e) Amtlich Topografisches Liegenschaftskataster (ATKIS) © Landesamt für Geoinformation und Landentwicklung Baden-Württemberg, License No.: 2851.9-1/19. www.lgl-bw.de.
f) GEOSTAT Switzerland; © Bundesamt für Statistik, Neuchâtel 2014, http://www.bfs.admin.ch/bfs/portal/de/index/dienstleistungen/geostat.html.
g) Landsat 5 Landcover classes: © LUBW (Landesanstalt für Umwelt, Messungen und Naturschutz Baden-Württemberg), download: http://www.lubw.baden-wuerttemberg.de/servlet/is/17328/.
h) Mire inventory, ©Bundesamt für Umwelt BAFU, download: http://www.bafu.admin.ch/schutzgebiete-inventare/07845/index.html?lang=de.
i) Forest Research Institute of Baden-Württemberg FVA, available upon request: http://www.fva-bw.de/monitoring/index9.html.
j) Switzerland: LiDAR; © Bundesamt für Landestopografie Swisstopo (DTM-AV, DOM-AV_ 2011) License No.: 5704 000 000, http://www.swisstopo.admin.ch.
k) Germany: LiDAR; © Landesamt für Geoinformation und Landentwicklung Baden-Württemberg, LGL, License No.: 2851.9-1/19, www.lgl-bw.de.
